# A Multiscale Model Evaluates Screening for Neoplasia in Barrett’s Esophagus

**DOI:** 10.1371/journal.pcbi.1004272

**Published:** 2015-05-22

**Authors:** Kit Curtius, William D. Hazelton, Jihyoun Jeon, E. Georg Luebeck

**Affiliations:** 1 Department of Applied Mathematics, University of Washington, Seattle, Washington, United States of America; 2 Program in Computational Biology, Fred Hutchinson Cancer Research Center, Seattle, Washington, United States of America; 3 Program in Biostatistics and Biomathematics, Fred Hutchinson Cancer Research Center, Seattle, Washington, United States of America; Indiana University, UNITED STATES

## Abstract

Barrett’s esophagus (BE) patients are routinely screened for high grade dysplasia (HGD) and esophageal adenocarcinoma (EAC) through endoscopic screening, during which multiple esophageal tissue samples are removed for histological analysis. We propose a computational method called the multistage clonal expansion for EAC (MSCE-EAC) screening model that is used for screening BE patients *in silico* to evaluate the effects of biopsy sampling, diagnostic sensitivity, and treatment on disease burden. Our framework seamlessly integrates relevant cell-level processes during EAC development with a spatial screening process to provide a clinically relevant model for detecting dysplastic and malignant clones within the crypt-structured BE tissue. With this computational approach, we retain spatio-temporal information about small, unobserved tissue lesions in BE that may remain undetected during biopsy-based screening but could be detected with high-resolution imaging. This allows evaluation of the efficacy and sensitivity of current screening protocols to detect neoplasia (dysplasia and early preclinical EAC) in the esophageal lining. We demonstrate the clinical utility of this model by predicting three important clinical outcomes: (1) the probability that small cancers are missed during biopsy-based screening, (2) the potential gains in neoplasia detection probabilities if screening occurred via high-resolution tomographic imaging, and (3) the efficacy of ablative treatments that result in the curative depletion of metaplastic and neoplastic cell populations in BE in terms of the long-term impact on reducing EAC incidence.

## Author Summary

Endoscopic screening for detecting cancer and cancer precursors in Barrett’s esophagus (BE) is currently informed by repeated systematic biopsying of the metaplastic BE tissue. Here we present a comprehensive multiscale model of the natural history of esophageal adenocarcinoma (EAC), which describes the entire multistage process beginning with the conversion event of normal squamous esophageal tissue to BE metaplasia, the spatio-temporal formation of independent dysplastic and malignant clones at the cell level, and finally the appearance of symptomatic EAC in BE. This model lends itself to a systematic exploration of the efficacy and sensitivity of current biopsy-based screening methods to detect neoplasia in BE patients, as well as alternative screening techniques based on high-resolution imaging of the BE tissue. Moreover, the model can also be used to predict the impact of ablative treatments on the risk of occurrence or recurrence of dysplasia or cancer. Due to the lack of studies that attempt to explicitly model the physical and biological dimensions of the screening process itself, our computational model provides a unique, publicly-available tool to improve understanding of factors that limit the efficacy of current screening protocols for BE patients.

This is a *PLOS Computational Biology* Methods paper.

## Introduction

The incidence of esophageal adenocarcinoma (EAC) has increased dramatically over the past few decades in the US and other Western countries, prompting numerous epidemiological and clinical studies to characterize etiologic, genetic, and environmental factors that may contribute to this alarming trend [1, 2]. EAC arises primarily (if not exclusively) in Barrett’s esophagus (BE), a metaplastic tissue alteration in the esophageal lining. Screening is targeted toward identifying BE patients who are at the highest risk of developing dysplasia and cancer. Although the risk of BE progressing to EAC is estimated to be low (around 0.2–0.5% per year [3]), clinical evidence suggests that the risk of neoplastic progression in BE varies significantly between individuals depending on age, gender, race/ethnicity, gastroesophageal reflux disease (GERD) and whether or not dysplasia is present in BE.

High grade dysplasia (HGD) occurring in BE is generally non-invasive but carries a high risk of progression to EAC. Low grade dysplasia (LGD) also occurs, but its clinical relevance is less certain. Most patients diagnosed with HGD undergo endoscopic mucosal resection (EMR) or treatment with radio frequency ablation (RFA) to remove HGD tissue and, in the case of RFA, to reduce the amount of underlying metaplastic BE tissue. Genetic and genomic studies, including longitudinal studies with multiple BE tissue samples from individual patients in the Seattle BE cohort [4], also implicate specific genomic alterations in the neoplastic progression process. Frequently observed alterations in BE include epigenetic silencing or loss of heterozygosity (LOH) of the *P16INK4A* and/or *TP53* tumor suppressor genes [5–8]. Whether these alterations necessarily lead to the clinical presentation of dysplasia and other cellular and architectural changes associated with this diagnosis is presently unknown. However, our working hypothesis is that fields of HGD are comprised of clonal populations of premalignant cells that originate from distinct progenitors in the BE tissue.

Because dysplasia (in particular HGD) continues to be a widely used clinical predictor for progression to EAC, most BE patients are recommended to undergo periodic endoscopic surveillance with biopsies taken at specified spatial locations in BE to detect neoplastic changes (dysplasia and/or cancer). However, due to the large number of adults with BE in the general population (∼ 1–3% [9, 10]), excessive or ineffective BE screening and surveillance that do not significantly reduce EAC incidence and mortality are a considerable public health concern.

To examine these issues, we developed a mathematical and computational framework that allows concurrent modeling of the BE-to-EAC progression and endoscopic screening for dysplasia and preclinical cancer prior to EAC diagnosis. We will present the screening model as three cohesive modules. First, we present the stochastic model for EAC at the cell level capturing key events of the random, GERD-dependent onset of BE, the initiation and stochastic growth of premalignant clones, malignant transformations in premalignant clones, and stochastic growth of malignant clones prior to (symptomatic) cancer detection. This framework provides a bridge between the cell and population scales and has previously been described for modeling EAC incidence data in the US [11, 12]. The following two, novel parts of the model utilize and improve on this prior work and are essential for describing the screening process.

The second module is an explicit computational method to efficiently simulate the entire cell model in an individual BE patient until the time of a hypothetical screen. This requires computation of the joint size distribution of premalignant and malignant clones in the BE tissue prior to development of an incident, symptomatic cancer. The method captures the clonal progression of an idealized, 2D *in silico* tissue composed of intestinal crypts and can generate a variety of spatial patterns (from circular to very diffuse shapes) of both premalignant and malignant clones within the BE segment of a patient.

The third module simulates an endoscopic screen of a patient’s BE segment. For a biopsy-based screen, the model mimics the Seattle standard protocol for screening patients with BE, probing the tissue every 1–2 cm with 4 quadrant biopsies for the presence of dysplasia and signs of invasive cancer. We show that the efficacy of this protocol is highly variable and dependent on the sensitivity of detecting neoplastic abnormalities within a biopsy. This sensitivity also affects the amount of dysplastic patients predicted to harbor undetected malignancy at time of screening. The outcomes of biopsy-based screens are then compared with the model’s prediction for screening outcomes when using high-resolution imaging, a new screening technology not yet widely in use. With information about the amount of small neoplasms that go undetected during biopsy-based screening, the model quantifies the potential advantages that image-based screening might offer. Finally, this module simulates ablative treatment of BE patients with detected dysplasia during screening. By explicitly modeling the curative effects of ablative treatment, we gain insights into the critical factors that may prevent treatment success.

## Methods

**Fig 1:**
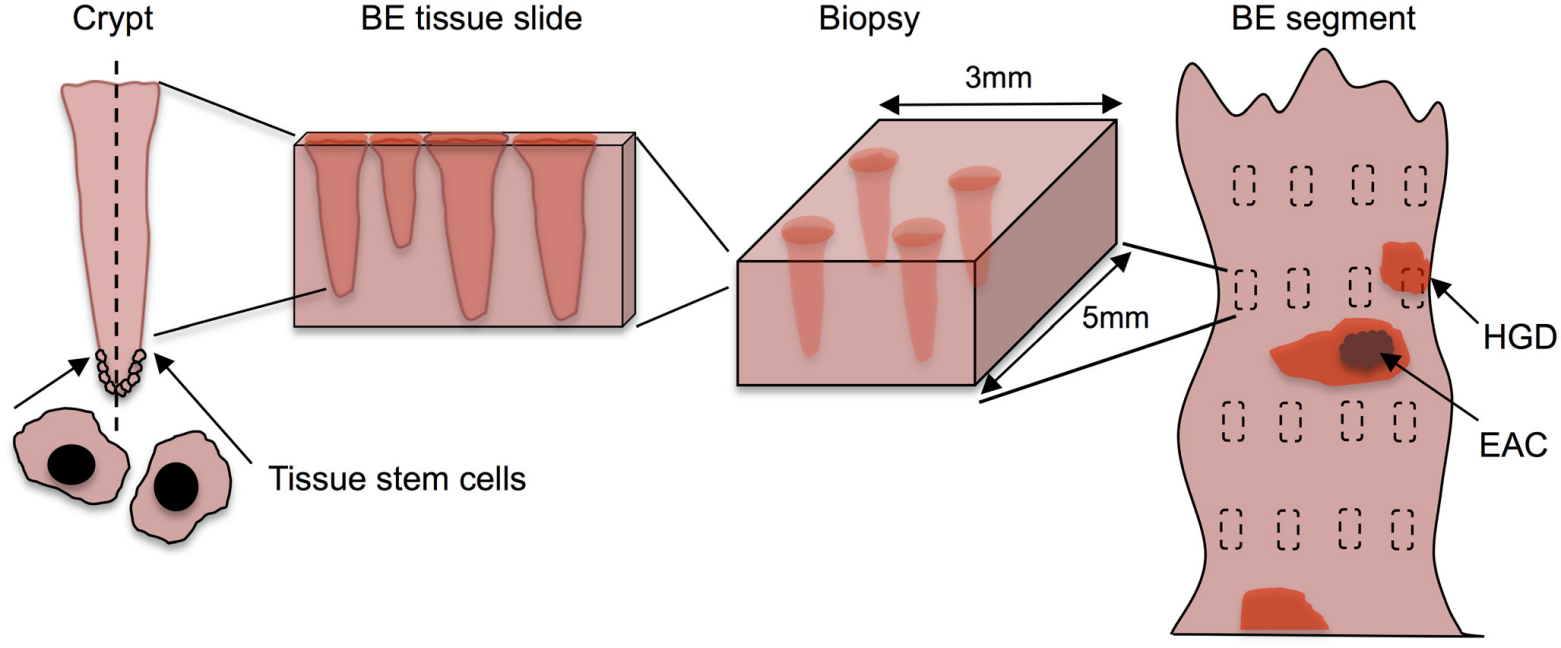
The multiscale nature of BE screening. Biopsy screening for BE scales from stem cells in the crypt (left) to the BE cylindrical segment of the esophagus depicted (right) with rectangles representing biopsy samples taken during endoscopy via the Seattle biopsy protocol. The BE segment may have dysplasia and/or malignant tissue patches that remain untouched after biopsying. During histological preparation, portions of each biopsy are sliced by microtome and placed on slides for pathologic assessment. Diagnosis is made by microscopic interpretation of crypt and cellular architecture, reflecting the most severe tissue grade found from all slides.

Here we describe the modular design of the multistage clonal expansion for EAC (MSCE-EAC) screening model. The first module provides a likelihood-based method for modeling the stochastic process of EAC formation used previously for model calibration to incidence data. The second module explicitly simulates the cell model outcomes and spatial organization of premalignant and malignant lesions in a Barrett’s esophagus (BE) segment. Lastly, the third module simulates an endoscopic screen, whether biopsy-based or image-based, at the tissue level to evaluate the number of BE patients who are positive for neoplasia. These latter tissue level modules require two new spatial parameters that can be calibrated to reproduce published screening prevalences of high grade dysplasia (HGD), and then used to predict other outcomes at the tissue and population scales. Fig 1 depicts each level of detail in our multiscale model for screening an individual BE patient: cell, tissue, and organ.

### MSCE-EAC Cell Module

The multistage clonal expansion for EAC (MSCE-EAC) cellular model assumes that the stepwise progression to cancer, formulated mathematically as a continuous-time Markov process, involves tissue alteration whereby part of the distal normal esophageal squamous epithelium (with variable extent) undergoes metaplastic transformation resulting in a columnar-lined epithelium called Barrett’s esophagus (BE). This tissue alteration provides a natural starting point for a cell-level description of the neoplastic progression to EAC.

Because gastroesophageal reflux disease (GERD) increases the risk of BE [13–15], we assume that the rate of conversion of normal esophageal tissue to BE metaplasia is GERD-dependent. Here we define symptomatic GERD (sGERD) patients as those with GERD symptoms occurring weekly or more frequently. This represents an extension of an earlier model that did not include the effects of GERD [16]. Specifically, we model the exponential BE rate, *ν*(*t*), as a function of the prevalence of symptomatic GERD at age *t*, such that

$$\begin{array}{c} \nu \left(t\right)=\nu_{0}\;(\left(1-p_{{}_{sGERD}}\left(t\right)\right)+RR\cdot p_{{}_{sGERD}}\left(t\right)), \end{array}$$ (1)

where *p*_*sGERD*_(*t*) is the prevalence of GERD symptoms at age *t* and *RR* is the relative risk *RR* of GERD for BE. The time-dependent cumulative distribution for BE onset is then given by

$$\begin{array}{c} F_{{}_{BE}}\left(t\right)=\mathrm{Pr}\left[T_{{}_{BE}}\leq t\right]=1-e^{-\int_{0}^{t}\nu \left(s\right)\;ds}. \end{array}$$ (2)

See S1 Text for details on modeling *p*_*sGERD*_(*t*) and S1 and S2 Figs for values of *p*_*sGERD*_(*t*) and BE prevalence, *F*_*BE*_(*t*), for males and females, respectively.

**Fig 2:**
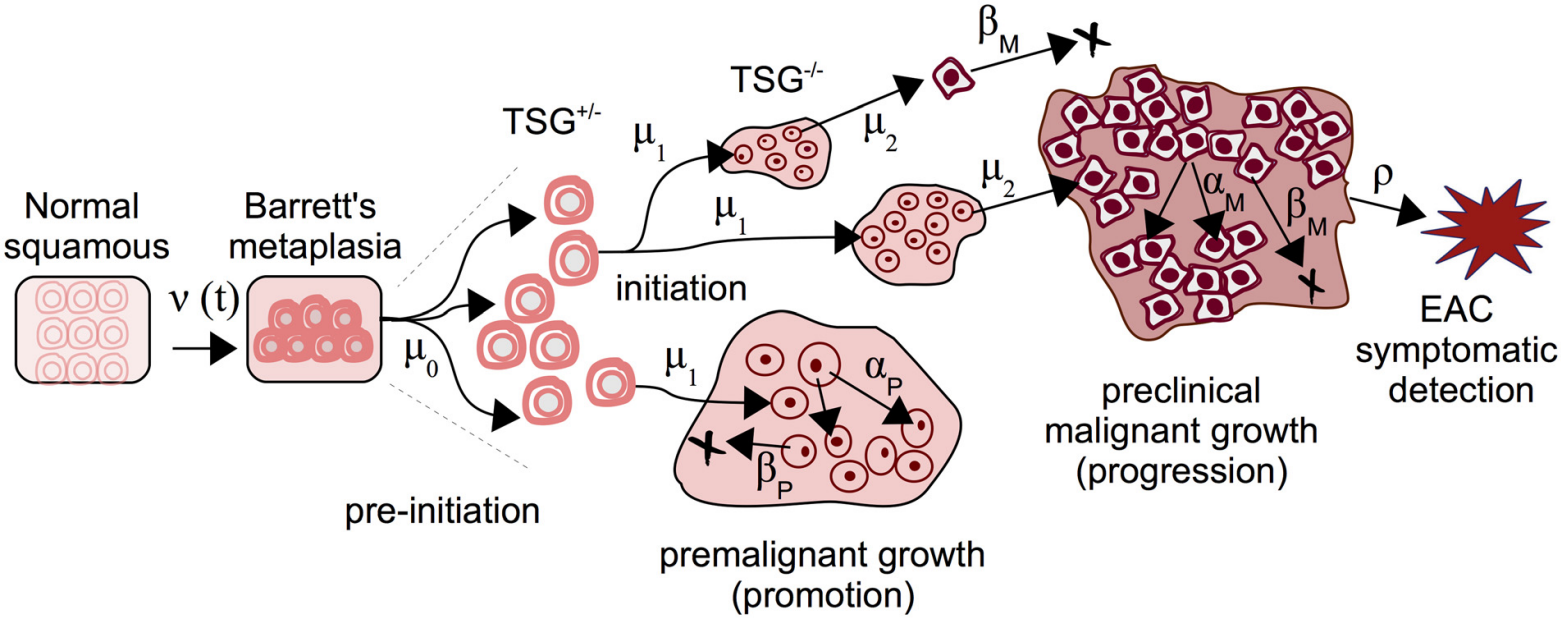
The multistage-clonal expansion model for EAC (MSCE-EAC) cell module. Normal squamous epithelium may transform to BE with an exponentially distributed onset time with rate *ν*(*t*), followed by a ‘two-hit’ tumor initiation process with Poisson initiation rates *μ*_0_, *μ*_1_, which leads to the stochastic appearance of premalignant progenitor cells in the tissue. Premalignant cells undergo a first clonal expansion described by a birth-death-migration process with cell division rate *α*_*P*_, cell death-or-differentiation rate *β*_*P*_, and malignant transformation rate *μ*_2_. Malignant cells, in turn, undergo a second clonal expansion by a birth-death-detection process with cell division and death rates *α*_*M*_ and *β*_*M*_, respectively, allowing for stochastic growth and possibly extinction of the malignant tumor. Clinical detection occurs through a size-based detection process with parameter *ρ*. TSG, tumor suppressor gene.

Once a tissue conversion occurs resulting in BE at exponentially distributed age *T*_*BE*_, the model continues as a multi-type branching process that includes stem cell counts of three different types: pre-initiated, initiated (premalignant), and malignant. This cellular description of carcinogenesis begins with the initiation of stem cells, enabling them to undergo clonal expansion. In the current formulation of the model, initiation occurs as a result of two rate-limiting events (e.g., bi-allelic inactivation of a tumor suppressor, such as *TP53*) due to previous likelihood-based model selection [16]. Once a stem cell is initiated, it undergoes clonal expansion through a stochastic birth-death-mutation (b-d-m) process with cell division rate *α*_*P*_ and cell death/differentiation rate *β*_*P*_, so thus *β*_*P*_/*α*_*P*_ is the asymptotic probability of extinction. An initiated (dysplastic) cell may also undergo a transforming mutation with rate *μ*_2_ that generates an initiated cell and a malignant cell. Malignant cells may undergo an independent clonal expansion with cell division and death rates *α*_*M*_ and *β*_*M*_, respectively, allowing for stochastic growth and possibly extinction of the malignant tumor. The inclusion of clinical, or symptomatic, detection of a malignant tumor occurs through a size-based detection process with parameter *ρ*. Thus, the model captures two distinct clonal populations of cells—premalignant (which we associate with dysplasia) and malignant (representing growth from early intramucosal to advanced carcinomas). See Fig 2 for an illustrated realization of this MSCE-EAC stochastic process.

This cell-level description is linked to the population scale by means of the model hazard function, defined as the instantaneous rate of detecting cancers among individuals who have not been previously diagnosed with cancer, as previously shown [12]. This quantity may be derived from the backward Kolmogorov equations for the stochastic multistage process described above and solved numerically via a system of coupled ordinary differential equations (ODEs) [17]. See S1 Text for a mathematical derivation of these equations and how the hazard function can be obtained from their solutions. Thus, one may infer rates of cellular processes from population level data as was previously done by likelihood-based calibration of the MSCE-EAC cellular kinetic parameters to incidence data, see [12] and S1 Text for more details. For the illustrations and examples presented in this paper, we use the parameter estimates for biological rates employed in [12], which are provided in Table 1.

**Table 1:** MSCE-EAC model biological parameters.

Value (95% CI)	Males	Females
*ν*_0_	3.65 (3.19–4.13) × 10^−4^	7.48 (4.87–10.29) × 10^−5^
*μ*_0_(*μ*_1_)	7.99 (6.38–9.83) × 10^−4^	7.05 (6.13–12.25) × 10^−4^
*μ*_2_	4.54 (3.65–6.47) × 10^−5^	6.89 (3.16–14.28) × 10^−5^
*g*_*P*,0_ *	9.91 (9.28–10.99) × 10^−2^	1.23 (1.06–1.35) × 10^−1^
*g*_1_ *	5.09 (2.75–5.90) × 10^−1^	6.40 (2.16–8.44) × 10^−1^
*g*_2_ *	5.38 (4.83–5.72) × 10^−2^	2.98 (2.47–3.44) × 10^−2^
*g*_3_ *	1912.5 (1909.1–1914.1)	1945.3 (1923.9–1954.4)

All parameter estimates fit to SEER incidence data and have the units of per cell per year. Markov Chain Monte Carlo 95% confidence intervals provided beside the maximum likelihood estimates.

*Parameters are elements of sigmoidal function for clonal proliferation (see S1 Text).

### MSCE-EAC Tissue Module

Our previous work with the stochastic, cell-level MSCE-EAC model did not directly calculate the expected number and sizes of independent focal lesions of each type in a patient’s BE segment at any given age in his/her lifetime, as depicted in Fig 2. However, this knowledge is clinically relevant for effectively monitoring progression to EAC in a BE patient. In this module, we first describe the computational tool developed to obtain the MSCE-EAC stochastic realizations of the number and sizes of premalignant and malignant lesions in a BE patient at any given age. Next, we use these model-derived outcomes of initiated stem cell numbers to simulate their spatial configuration as lesions in the BE tissue, which is important given the spatial nature of the biopsy screening protocols.

Because the mathematical complexity of this multistage model makes it difficult to derive tractable analytic size distributions for all cell types through time we resort to direct simulations of sample populations of individual lifetime trajectories to track clone number and sizes as progeny from certain cell types. Recent advances in stochastic simulation allow further efficiency in computation of cell counts, enabling rapid model testing and examination of many possible scenarios. See S1 Text for the full algorithm and implementation of the MSCE-EAC hybrid simulation of the number of clones and their sizes for all cell types present at time *t*_*s*_ during a hypothetical screening. We call this a ‘hybrid’ simulation because it employs stochastic simulation when necessary but also makes use of samples from analytical distributions when possible. For the simulation of premalignant (dysplastic) clones, we employ two methods. The first is an exact method, the stochastic simulation algorithm (SSA), first described by Gillespie [18], that simulates every jump in cell count and exponential waiting times between events. The second is a highly efficient approximation to SSA called *τ*-leaping. S1 Text explains these two methods and describes how the MSCE-EAC simulation uses them cooperatively in a highly efficient approach. The accuracy of both the size distributions generated by the SSA and the *τ*-leaping method are shown in S3 Fig as Q-Q plots for the size distributions of non-extinct premalignant clones compared to the analytical distribution for an independent b-d-m process.

**Fig 3:**
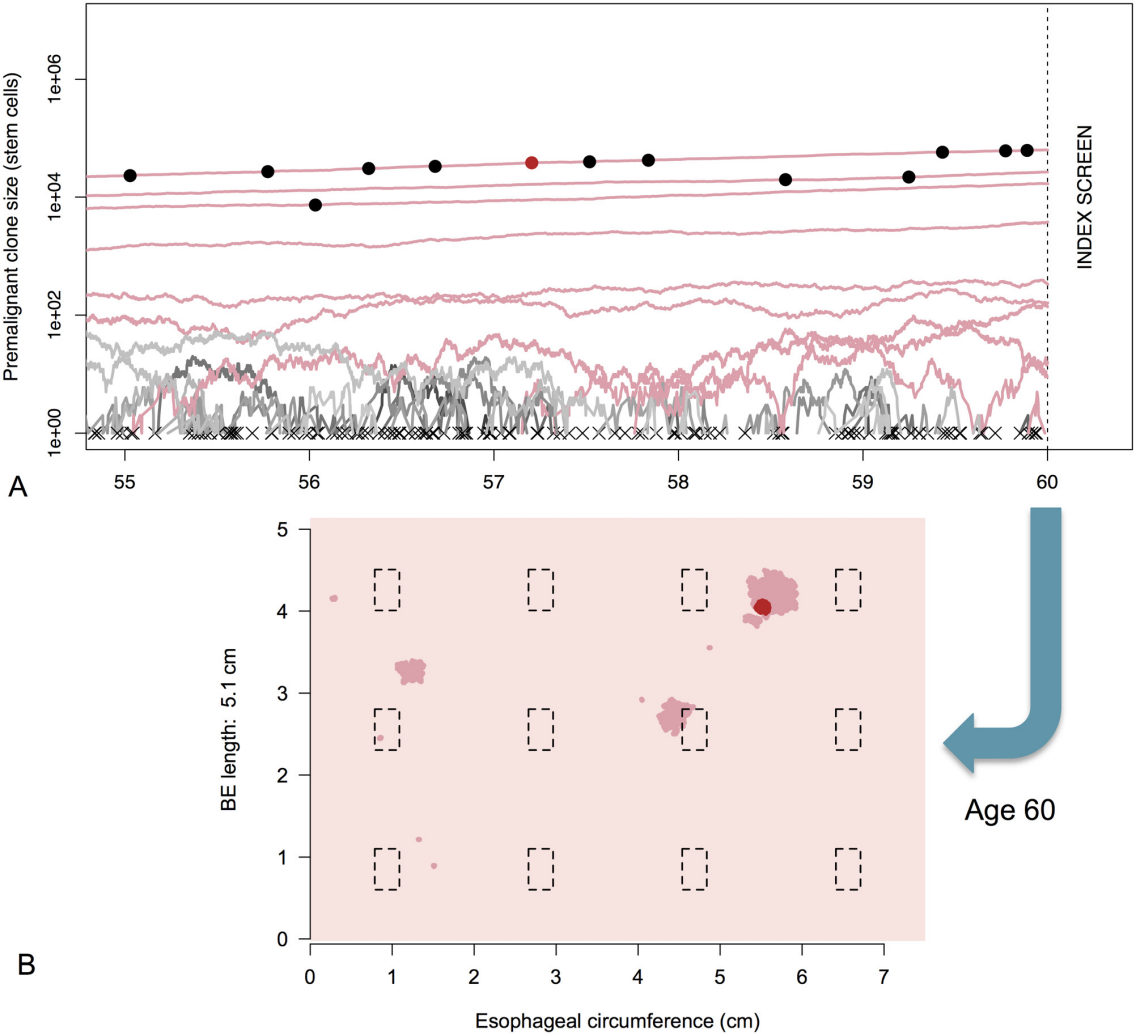
MSCE-EAC tissue module. A) Premalignant (dysplastic) clone growth stochastic trajectories are depicted for a sample male BE patient from the 1930 birth cohort, beginning five years before time of initial screen at age 60. Most dysplastic cell progenies from a single initiated cell went extinct before examination by endoscopy (trajectories depicted by gray lines and an ‘x’ at time of extinction). The dysplastic clone trajectories that did not go extinct before index screen are shown in dark pink with final sizes intersecting the vertical dotted line. The dots on trajectory lines correspond to times of asymmetric division of a dysplastic cell to produce one dysplastic cell and one malignant cell. Malignant transformations produce both clones that quickly went extinct before age 60 (black dots) and non-extinct malignancies (red dot). B) BE segment at time of screening. Same simulated male patient from (A) with BE of length 5.1 cm. Dysplastic clones (dark pink), malignant clone (red), and biopsies (black dashed rectangles) are pictured at time of biopsy-based screening, age 60. Clone diffusivity parameter is *γ* = −2.

With cell module parameters as input, the MSCE-EAC hybrid algorithm simulates the multi-type branching process for an individual’s cellular progression from birth until time (age) *t*_*s*_, which can be repeated to generate (synthetic) data for a sample population. In summary, for those individuals who are found to have BE by time of screening, each patient has a specific number of BE stem cells (*X*), number of pre-initiated cells (P*), a number of non-extinct premalignant (*P*) clones with respective sizes, a number of non-extinct malignant (*M*) clones with respective sizes and information about the parental *P* clones from which the *M* clones originated, and lastly whether the patient is a prevalent, clinical EAC case by time *t*_*s*_. Note, the stochastic model captures the possibility that the ancestor premalignant clone may go extinct while the malignant clone is still growing at the time of screening *t*_*s*_. Fig 3A shows the random trajectories for a simulated BE patient’s clones obtained via this algorithm for the five years of life prior to initial screening at age *t*_*s*_ = 60.

#### Crypt-structured spatial modeling of neoplasia

Thus far, the MSCE-EAC tissue module has employed the cell-level rate parameters, described and fitted to population data in the MSCE-EAC cell module, to obtain realizations of the multi-type branching process for a BE patient until time of screening. In this section, we translate the size, in terms of the number of stem cells, in each neoplastic lesion simulated by the hybrid algorithm to the actual 3D size of the neoplasm within the BE segment, which is important for predicting what is detected during an endoscopic screen. This translation requires us to consider the known spatial characteristics of BE tissue biology. The metaplasia that defines BE is made up of a monolayer of epithelial cells that form crypt-like depressions in the underlying connective tissue of the lamina propria that are considered the basic functional unit of BE tissue [19]. These units (or *crypts*) are renewed through asymmetric divisions that produce differentiated cells, which eventually die or are sloughed off the surface epithelium. Putative stem cells found at or near the crypt base appear to escape this flow. Thus, under homeostatic conditions, the stem cell populations are assumed to remain immortal in the tissue before initiation of dysplasia. However, once a stem cell is initiated (which required two-rate limiting events in the MSCE-EAC model), it may undergo stochastic clonal expansion through symmetric divisions that produce two identical initiated stem cells or two cells committed to differentiation, or it may undergo cell death (apoptosis).

Although the mechanisms regulating neoplastic growth across crypt structured tissues are not well understood [20], it is generally assumed that initiated stem cells either spill over and invade neighboring crypts (“top-down” hypothesis) or that bifurcation of crypts may occur in a process called *crypt fission* that leads to clonal expansion at the crypt level (“bottom-up” hypothesis) [20]. Recent studies favor clonal expansion by crypt fission as the dominant mode of generating mutated crypts in the human colon that form adenomas [20]. Our computational model includes the assumption that crypt fission drives the spatial growth of neoplastic lesions in BE.

Due to this spatial structure of crypt openings at the epithelial surface, we can represent the neoplastic clones on a 2D plane even though the crypts in BE constitute a 3D tissue as depicted in Fig 1. We introduce a single adjustable parameter for stem cell area density, *σ*, which equals the number of stem cells per mm^2^. To translate this parameter into numbers highlighting the cryptal organization of the BE segment, this area density can be defined as *σ* = *c*_*σ*_ ⋅ *k*, where *c*_*σ*_ is the number of crypts per mm^2^ and *k* is the number of BE stem cells maintaining each crypt. Although *σ* is the parameter we will vary in our model, we introduce this representation because experimental estimates are reported in terms of crypts and stem cells in each crypt. Estimates for the crypt density *c*_*σ*_ in BE epithelium appear to show large inter-individual variation. For example, a recent study following a cohort of BE patients found *c*_*σ*_ to range from 3 to 100 (10,000–400,000 crypts per ∼ 38cm^2^) [21]. The cause and significance of this variation remains unclear.

Similarly, the stem cell population size *k* maintaining the crypt’s stem cell niche is also highly uncertain according to estimates provided in the literature on epithelial crypts. For colonic crypts, Nicolas and colleagues estimated there are between 8 and 20 stem cells per crypt [22], yet earlier methylation studies reported ∼ 64 per crypt [23]. There is also evidence that stem cell numbers may be significantly higher in dysplastic crypts in the colon [24]. Combined, these estimates of *c*_*σ*_ and *k* provide us with an estimated range of *σ* ∈ [24, 6400]. Because of the high experimental uncertainty for the parameter *σ*, we allow this to be a variable parameter in our model (see Results for our calibration of *σ*).

To generate the spatial representation of the clones, each premalignant clone is randomly placed on a hexagonal grid representing the crypt-structured BE tissue. While the overall area occupied by a clone is controlled by the number of stem cells it contains and the stem cell density *σ*, the spatial appearance of the clones is independently controlled by a diffusivity parameter *γ*. Briefly, clones are compact when *γ* > 0, becoming nearly circular when *γ* approaches 1. Alternatively, clones are made increasingly more diffuse and branching when *γ* < 0. Like *σ*, the spatial irregularity of neoplasm growth in BE tissue is not well known. Thus, like *σ*, we will consider *γ* as a second spatial variable parameter in our model.

Under our model, any premalignant ancestor cell may generate a neoplastic clone including a simulated number of premalignant cells and possibly an embedded population of independently expanding malignant cells that originate from malignant transformation(s) with rate *μ*_2_. To accommodate malignant tumor growth, we assume that malignant crypts simply displace the pre-existing premalignant crypts, a circumstance that is often seen in esophagectomy specimens [25].

In summary, for each BE patient, the MSCE-EAC tissue module first obtains the number and sizes of both premalignant and malignant clones (at time *t*_*s*_) randomly generated by the MSCE-EAC hybrid simulation and then translates these numbers to a spatial configuration of neoplasms within a patient’s BE segment. To illustrate this, we use the simulated patient’s cellular information provided from MSCE-EAC simulation shown in Fig 3A, and show in Fig 3B the corresponding ‘rolled out’ BE cylindrical segment generated with the spatial representation of the clones as described above, using the choices of *σ* = 3300 stem cells/mm^2^ and *γ* = −2. Simulated premalignant and malignant lesions are depicted in dark pink and red, respectively. The black, dotted line rectangles represent the location of biopsies taken from the tissue under a certain screening protocol that we will explain in the following section.

### MSCE-EAC Screening Module

The MSCE-EAC tissue module computes the number of stem cells in each neoplastic clone and generates the shapes of these clones within a BE segment at any given age of a patient. The MSCE-EAC screening module takes this information and performs an endoscopic screen on this realized BE segment. Here we outline the methodology for generating model predictions related to three specific screening outcomes: (1) the probability that small cancers are missed during biopsy-based screening, (2) the potential gains in neoplasia detection probabilities if screening occurred via high-resolution tomographic imaging, and (3) the efficacy of ablative treatments that result in the curative depletion of metaplastic and neoplastic cell populations in BE in terms of the long-term impact on reducing EAC incidence. These model predictions are described in Results.

#### Biopsy screens and diagnostic sensitivity

In 1998 the American College of Gastroenterology (ACG) recommended the use of a systematic sampling method known as the Seattle biopsy protocol rather than random biopsy sampling of BE tissue during endoscopic screening. This systematic biopsy protocol specifies 4 jumbo quadrant biopsies (∼ 15mm^2^ of tissue each) every 1–2 cm of the BE length to achieve increased sensitivity for detection of dysplasia [26–28]. However, a multi-center study from 34 US states on BE surveillance found that adherence to guidelines was only seen in 51.2% of cases [29]. The authors also found that longer segment BE, which is more time-consuming to biopsy, was significantly associated with reduced adherence even though risk of EAC is considered proportional to the length of the BE segment. In the examples described here, we model endoscopic screening performed by the Seattle protocol, although the methods may be applied to any protocol of biopsy placement and/or biopsy size during upper endoscopy. Therefore, the model may be utilized for comparing simulated efficacies of different screening protocols. As we will see in the scenarios that follow, for an average esophageal circumference of 75 mm, even the rigorous Seattle biopsy protocol results in large sampling errors due to the fact that biopsies only sample 4–6% of the total BE mucosal surface for pathologic assessment.

Nearly half of BE surveillance endoscopies in the US are not performed in adherence to the Seattle biopsy protocol [29] because less tissue is actually sampled than prescribed due to use of smaller forceps or fewer biopsies taken. Specimens for histology are typically fixed in formalin, embedded in paraffin, and tissue profiles are cut at 4–5*μm* and stained with haematoxylin and eosin (H&E) [25], but only few histological slides are actually examined for neoplastic changes per biopsy and the precise method of examining specimens is usually not described in publications. All of the aforementioned factors may account for the large range of estimates found for prevalences of dysplasia and/or intramucosal cancer [25, 30–34].

To account for different biopsy protocols, incompletely described histological methods, and interobserver variation of neoplasia grade, we present results from the computational model for different levels of diagnostic sensitivity based on the minimum number of neoplastic (premalignant/malignant) crypts within a simulated biopsy specimen required for pathologic diagnosis of dysplasia/malignancy among BE patients without prior diagnosis of EAC. As depicted in the simulated BE segment of Fig 3B, some biopsy specimens taken via the Seattle protocol contain varying numbers of neoplastic crypts while missing sizable neoplastic areas or even entire neoplastic clones. Since the pathologist usually assesses less than 1/10 of the actual specimen after histologic sectioning, there is high probability that, even if neoplastic crypts exist in a biopsy specimen, no neoplastic crypts are contained on a histologic slide after sectioning. As a way to capture this sensitivity, we estimated prevalences of neoplastic tissue for a range of biopsy thresholds, i.e., the minimal proportion of neoplastic stem cells in a single biopsy needed for the neoplasm to be detectable at time *t*_*s*_. Let *n*_*f*_ be the fraction of neoplastic stem cells present out of the total number of BE cells within a single biopsy that are required for a positive diagnosis of neoplasia. Recall that the spatial parameter *σ* defines the relationship between numbers of cells in each clone and geometric clone size. As an example, if *n*_*f*_ = 1/2, then a simulated patient must have at least one biopsy (among all of the biopsies obtained via the Seattle protocol) that contains at least $\frac{15}{2}\sigma$ neoplastic cells (50% of stem cells in a single 15mm^2^ biopsy) for a positive diagnosis of neoplasia. Therefore, the *biopsy sensitivity* of a screen simulated by the MSCE-EAC screening model is interpreted as (1 − *n*_*f*_) ⋅ 100%.

#### High-resolution imaging screens

Beyond different biopsy-based protocols, the screening module within the MSCE-EAC screening model allows the user to also choose to simulate any screen using high-resolution imaging within the esophagus. The model can perform an optical coherence tomography (OCT) screen in which a positive detection of HGD and/or malignancy occurs if the geometric size of a clone on image is greater than a resolution area threshold, *a*_*OCT*_. Thus, we no longer need to consider neoplastic proportion thresholds as we explored previously for biopsy-based screening, but rather consider a fixed area or caliber threshold of any imaged clone. The results of an imaged-based screen will again depend on parameter *σ* to determine if the geometric size of a clone is above or below an imaging resolution.

#### Ablative treatment and impact on EAC incidence

After a simulated screen of BE patients for detection of dysplasia and preclinical EAC at age *t*_*s*_, the MSCE-EAC screening module can be used to further simulate an intervention such as an ablative treatment using radio frequency. To replicate current practice with radio frequency ablation (RFA), we simulate RFA treatment on positively screened, non-EAC patients with dysplasia. The MSCE-EAC screening module then projects the EAC incidence into the future after an ablative treatment. Ablation is assumed to curatively reduce all clonal populations and the number of BE crypts by certain percentages. As a simple example, we consider the model’s EAC incidence predictions after a single ablative treatment when indicated by the presence of high grade dysplasia on future EAC incidence.

For all times *t* > 0, we can compute the cumulative hazard Λ_*MSCE*_(*t*),

$$\Lambda_{{}_{MSCE}}\left(t\right)=-\ln\left(S_{{}_{MSCE}}\left(t\right)\right)=-\ln(1-\int_{0}^{t}f_{{}_{MSCE}}\left(s\right)\;ds)$$ (3)

where *T*_*C*_ is the random variable for EAC cancer detection and *f*_*MSCE*_(*s*) is the corresponding MSCE-EAC density function. For the initial scenario of screening all individuals at time *t*_*s*_, we derived the MSCE-EAC cumulative hazard function that includes contributions from the subpopulation of individuals found to have BE at time *t*_*s*_ who, immediately following HGD diagnosis, receive treatment at time *t*_*s*_; and the subpopulation without BE. For any time *t* > *t*_*s*_ and BE cumulative distribution *F*_*BE*_ given in Eq (2), we compute *f*_*MSCE*_(*s*) as follows

$$f_{{}_{MSCE}}\left(s\right)=f_{{}_{MSCE}}\left(s|T_{{}_{BE}}\leq t_{s}\right)\cdot \mathrm{Pr}\left[T_{{}_{BE}}\leq t_{s}\right]+f_{{}_{MSCE}}\left(s|T_{{}_{BE}}>t_{s}\right)\cdot \mathrm{Pr}\left[T_{{}_{BE}}>t_{s}\right].$$ (4)

For the screened BE population we follow the method of Jeon et al. [35] to simulate the four possible types of cells present in a patient at screening time $t_{s}^{-}$ (where the minus superscript denotes cell populations present prior to any intervention): *X* = number of BE stem cells in the BE segment, $P^{*}(t_{s}^{-})=$ number of preinitiated P* cells, $P(t_{s}^{-})=$ number of initiated, dysplastic *P* cells (all clones combined), $M(t_{s}^{-})=$ number of malignant, preclinical cancer cells (all clones combined). The MSCE-EAC tissue module simulates realizations of these random variables for each patient up to the instance of screening $t_{s}^{-}$, before intervention occurs. After simulating *n* independent and identically distributed realizations of individuals (by gender) and performing the Seattle biopsy screening protocol *in silico* on those with BE as described earlier, the screening module provides the vector $A_{i}=\{X_{i},P_{i}^{*}(t_{s}^{-}),P_{i}(t_{s}^{-}),M_{i}(t_{s}^{-})\}$ for each patient *i* with BE, *i* = 1, …, *n*.

Next, RFA intervention may be simulated for patients diagnosed with dysplasia by introducing the following *ablation proportion* vector, *ω* = {*ω*_*X*_, *ω*_P*_, *ω*_*P*_, *ω*_*M*_}, that describes the cell-type specific depletion of BE tissue. For example, to simulate a perfect ablation of all lesions in BE and of BE metaplasia itself, we set *ω* = {0, 0, 0, 0}. For those patients who have a positive screen, we simulate an RFA treatment by adjusting the patient’s (simulated) cell count vector *A*_*i*_ through component-wise multiplication by *ω*. Thus, the post-RFA numbers of cells in each stage of the MSCE process immediately after screening and treatment (denoted by time $t_{s}^{+}$) are given by the adjusted cell type vector ${\hat{A}}_{i}$

$$\begin{array}{c} {\hat{A}}_{i}\equiv \omega οA_{i}=\left\{\omega_{{}_{X}}\cdot X_{i},\omega_{{}_{P^{*}}}\cdot P_{i}^{*}\left(t_{s}^{-}\right),\omega_{{}_{P}}\cdot P_{i}\left(t_{s}^{-}\right),\omega_{{}_{M}}\cdot M_{i}\left(t_{s}^{-}\right)\right\} \end{array}$$ (5)

$$\begin{array}{c} =\{X_{i}\left(t_{s}^{+}\right),P_{i}^{*}\left(t_{s}^{+}\right),P_{i}\left(t_{s}^{+}\right),M_{i}\left(t_{s}^{+}\right)\}. \end{array}$$ (6)

BE patients with a negative screen for neoplasia sustain the same (before and after) $A_{i}\equiv {\hat{A}}_{i}$ vector as was computed at time $t_{s}^{-}$ since no RFA treatment is performed on these patients. Due to the Markovian renewal property of branching processes, the survival and hazard functions for each screened patient *i* = 1, …, *n* for some time *t* > *t*_*s*_ are computed using the adjusted numbers for each cell type post screen. These survival and hazard functions for the 4-stage model after BE onset are easily computed using the Kolmogorov backward equations for the stochastic multistage process. See S1 Text for the full derivation of all individual contributions to Λ_*MSCE*_(*t*) defined by Eq (3).

### Open Source Code

The methods outlined in this section are implemented by the comprehensive MSCE-EAC screening model consisting of three modules: cell, tissue, and screening. All necessary tools to employ this method, including examples of user inputs used in the upcoming Results, are available in documented R code at https://github.com/yosoykit/MSCE_EAC_Screening_Model.

## Results

Clinical studies that assess the efficacy of screening Barrett’s esophagus (BE) patients are naturally limited by the amount of BE tissue that can be sampled for histopathological analysis. To gain insights into how this limitation affects screening efficacy we used the described EAC multiscale method to compute the unobserved proportion of precursor lesions and early cancers, quantities of clinical relevance for early cancer detection. As shown here, the multistage clonal expansion for EAC (MSCE-EAC) screening model predicts all lesions in BE during a patient’s lifetime, including their numbers and sizes. The explicit calculation of the numbers of stem cells in these lesions is functionally dependent on the model parameters that, with the exception of the spatial parameters introduced the Methods section, were previously estimated through model calibrations to EAC incidence data in the US [12]. For each simulated BE patient, we performed a pre-specified screening protocol to ascertain a patient’s clinical diagnosis, while also retaining concurrent information on any undiagnosed, potentially detectable lesions.

In the following results, we first calibrated the two unknown spatial parameters (neoplastic lesion shape diffusivity parameter *γ* and stem cell density parameter *σ*) to achieve consistency with current literature findings on the prevalence of the most important neoplastic precursor, high grade dysplasia (HGD), in BE patients, without changing the parameters that determine the fits to EAC incidence data reported in [12]. Thus fully calibrated, we then applied the MSCE-EAC screening model to predict three important clinical outcomes, including biopsy and imaging diagnostic sensitivities and the impact of ablative treatment on the risk of developing EAC.

### Calibration to High Grade Dysplasia Prevalence Data

In the current epidemiological literature, studies beginning with a biopsy-based index screen of BE patients (i.e., the screen when a patient is first diagnosed with BE) provide widely variable estimates of the prevalence of HGD, ranging from ∼ 2.75–8.25% [25, 30–34]. To compare this with model-derived predictions, we simulated an index endoscopic screen on a sample population of patients with BE and computed the prevalences of both premalignancy (HGD) and screen-detected (non symptomatic) malignancy. For an illustrative example of the MSCE-EAC screening model outputs, we simulated an index endoscopy for all males and females at screening time (age) *t*_*s*_ = 60 in the year 1990 (indicative of index screens from prospective studies that estimate the BE to EAC progression rate). With the BE prevalence given in Eq (2), these results focus on expected observations in output regarding the subpopulation of individuals found with BE, for whom the MSCE-EAC screening model provides screening results (see Methods).

**Fig 4:**
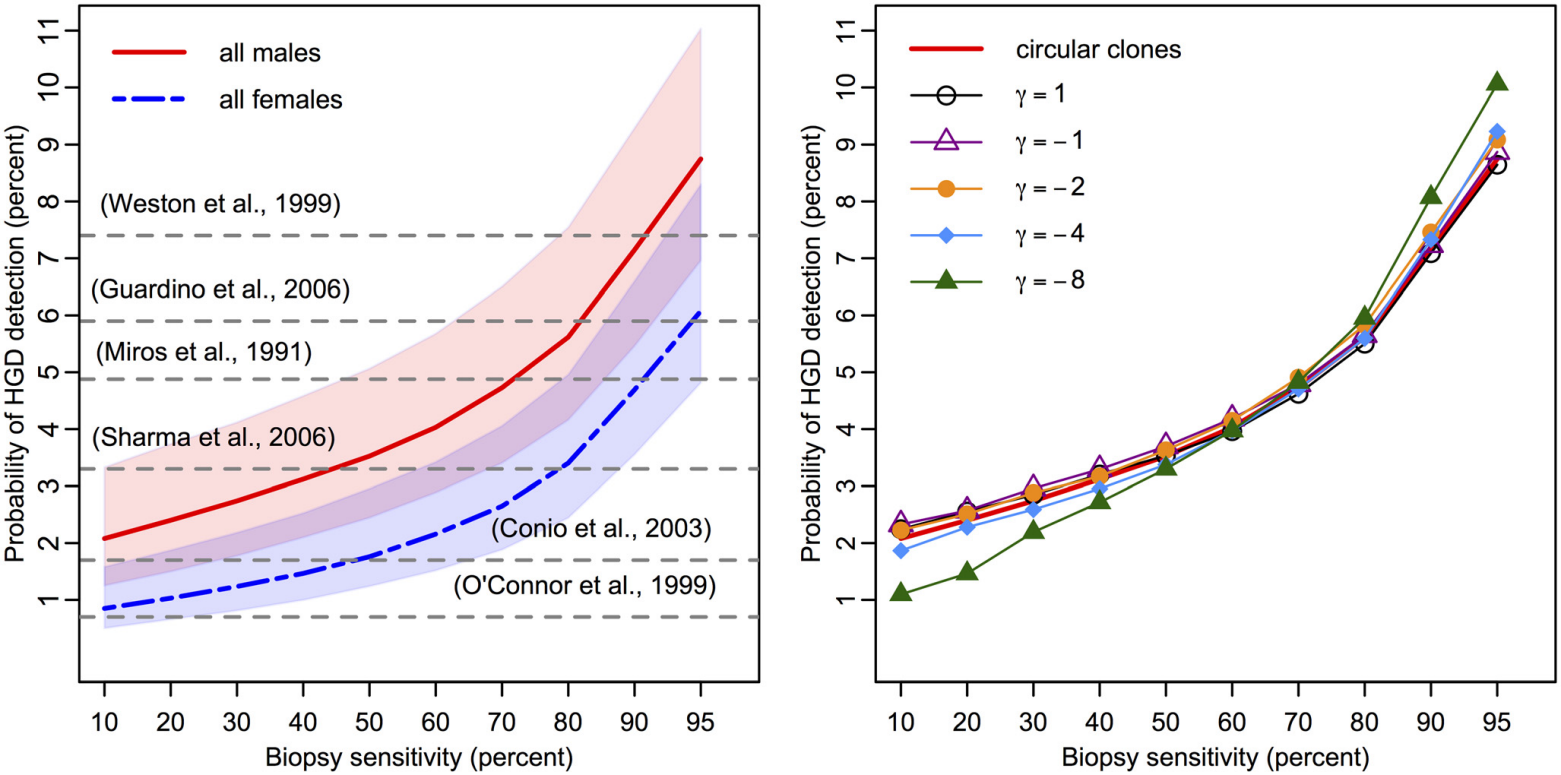
High grade dysplasia prevalences in BE estimated with the MSCE-EAC screening model. (Left panel) Probability of high grade dysplasia detection among BE patients simulated by the MSCE-EAC screening model for males (red, solid) and females (blue, dash-dotted) at initial screen at age 60 for biopsy sensitivities ranging from 10%–95% and assumed density of *σ* = 3300 stem cells/mm^2^ (shaded regions represent sensitivity of results for *σ* ∈ [2000, 5000]). Since the sensitivity of each study is unknown, literature values for the corresponding probability of HGD detection are depicted as horizontal grey dotted lines at a single percentage level [25, 30–34]. Expected prevalences produced by 100K simulation size of BE patients, shown for males and females. Simulation standard error is less than .001 for all Results. (Right panel) Male HGD prevalences produced by diffusive clone growth on hexagonal BE grid, for diffusivity parameter *γ* ranging from 1 to −8, versus isotropic, circular clone assumption (red, solid). Solid curve is the same as that shown for males in left panel. Probabilities of finding HGD for spatially simulated diffusive clone growth are shown for *σ* = 3300 stem cells/mm^2^ and identical biopsy sensitivities as shown in left panel. As explained in Methods, the assumption of *γ* = 1 results in almost identical prevalences to the circular clonal growth assumption. Overall, the results from 10K simulated male BE patients yield very similar prevalences regardless of clone shape for mid-range biopsy sensitivities.

**Fig 5:**
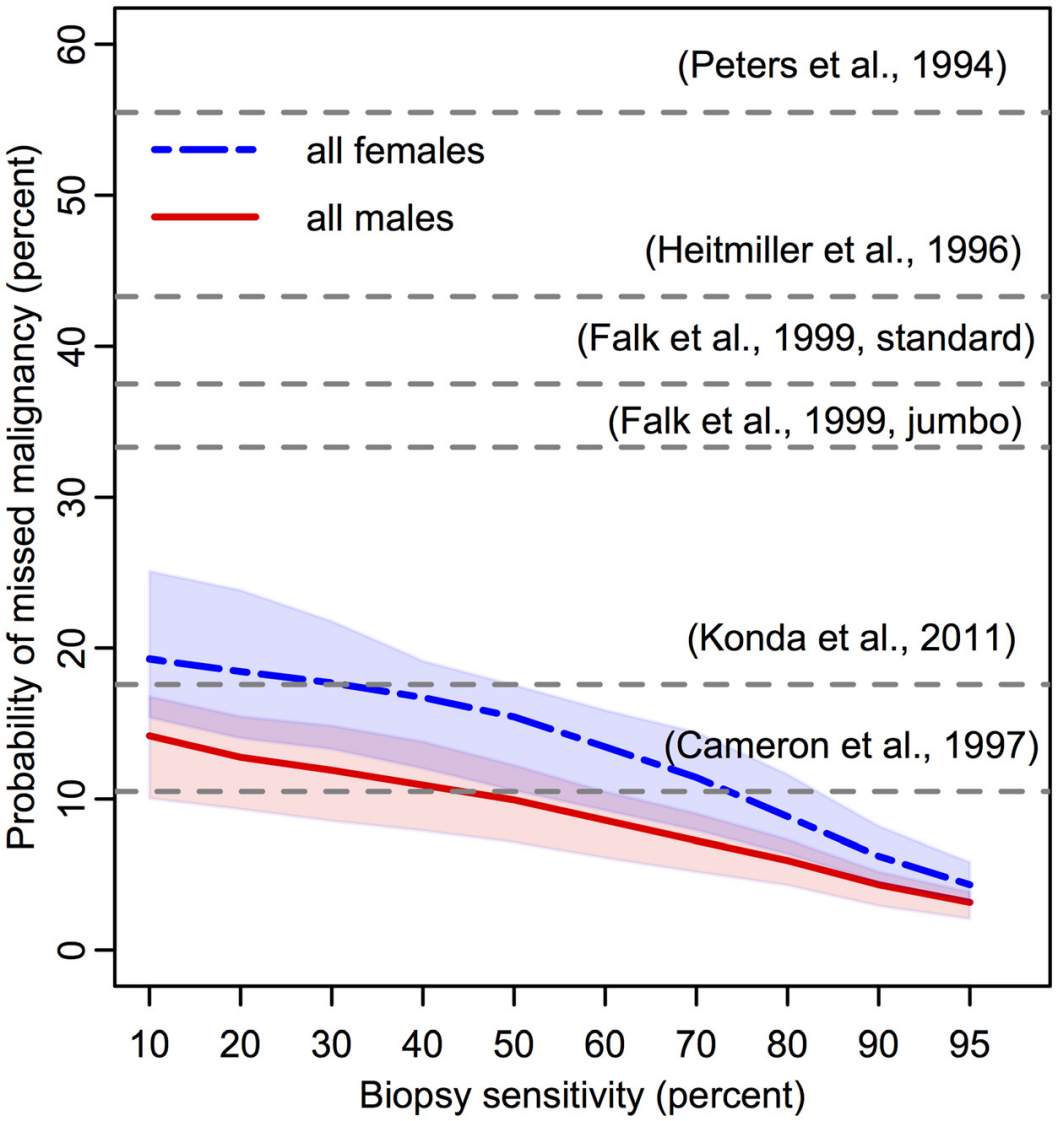
Predicted probability of missed malignancy in positive high grade dysplasia population at index screen. Percentages of patients diagnosed with HGD during index endoscopy (denominator is population plotted in Fig 4) who concurrently harbored missed, malignant clone(s) present on their BE segments that were not detected on biopsy screen. Since the sensitivity of each study is unknown, literature values for the corresponding probability of missed malignancy are depicted as horizontal grey dotted lines at a single percentage level [25, 30–34]. One publication included percentage of occult malignancy after HGD was diagnosed with either standard or jumbo forcep sizes, as indicated [38]. Expected proportions produced with 100K BE patient simulations each for males (red, solid) and females (blue, dash-dotted) with assumed density of *σ* = 3300 stem cells/mm^2^ (shaded regions represent sensitivity of results for *σ* ∈ [2000, 5000]).

Because the detection of a neoplastic lesion may involve both premalignant and malignant cells transformed within the lesion, we first consider the (random) sum of the two cell types to determine the efficacy of the biopsy protocol to detect a neoplastic lesion in BE. The biopsy sensitivity was varied from 10% to 95%, as seen in Figs 4 and 5, to allow for systematic exploration of sensitivity effects (see Methods). If a neoplasm is detected on a biopsy, we doubled the biopsy sensitivity for malignant content because the biopsy is under closer inspection.

#### Parameter estimation and sensitivity

To compute the prevalence of HGD without concurrent malignancy, we adjusted the BE population denominator to not include those with screen-detected cancer on index endoscopy. We then computed the probability of detecting HGD for a range of values for *σ* and *γ*. We found that when *σ* is assumed to be in the range of 2000 to 5000 mm^2^, the model produces HGD prevalence estimates that are broadly consistent with the range of published estimates for the span of biopsy sensitivities (see Fig 4) [25, 30–34]. In particular, the model estimated an expected range of 2.1%–8.7% for HGD prevalence among men and an expected range of .85%–6.1% for HGD prevalence among women when using a density of *σ* = 3300 stem cells/mm^2^. For each choice of *σ*, the results were mostly insensitive to the choice of diffusivity parameter *γ* (see right panel of Fig 4).

We present forthcoming results using the cell-level parameters found in Table 1, *σ* = 3300 (*σ* ∈ [2000, 5000] shown for sensitivity illustration), and *γ* = 1. The model parameters reproduce population-level EAC incidence data (obtained through symptomatic detection [12]) and HGD prevalence data (obtained from biopsying BE tissue at specific locations using a standard protocol).

By estimating the same breadth of HGD prevalences as studies have reported [25, 30–34], Fig 4 illustrates our prediction that HGD detection is strongly dependent on *n*_*f*_, the minimum neoplastic tissue fraction for detection in a single biopsy (see Methods). Specifically, by increasing biopsy sensitivity from 10% to 95%, our model predicted that the prevalence of HGD increases 4-fold. Further, even though most clones remain undetected, the mean number of non-extinct premalignant clones in this cohort was 6.6 per BE patient (See S4–S7 Figs). This modeling result highlights the prediction that even with rigorous adherence to the Seattle protocol, the majority of BE patients with concurrent HGD will not be detected nor considered prevalent HGD cases.

### Missed EAC Malignancies in HGD Patients

Along with difficulties in first detecting dyplasia present in BE during endoscopic screening, several studies suggest that many BE patients who are diagnosed with HGD without malignancy actually have an undetected cancer that was missed during biopsy screening [36, 37]. The MSCE-EAC screening model estimates the probability that a positive HGD patient actually harbors a synchronous, occult malignant clone that is not screen-detected either because it was completely missed in a biopsy sample (e.g. see the small malignancy depicted in Fig 3B) or because it was undetected in a biopsy for a particular biopsy sensitivity, perhaps due to insufficient histologic sectioning. This is an interesting, clinically relevant feature of our modeling. The model predicted the expected fraction of undetected EAC in BE patients diagnosed with HGD to be between 3.2%–14.2% for men and 4.3%–19.3% for women (see Fig 5). We conclude that the higher probability of missed malignancy in women is due to the lower probability of finding any neoplasia (due to smaller clone sizes, S4–S7 Figs) in women during index endoscopy (see Fig 4).

These predicted ranges are compared with studies of HGD patients found with concurrent adenocarcinoma, which remained undetected even by rigorous biopsy protocols but are later discovered during resection of the esophagus [38–42]. However, from these esophagectomy studies conducted over the past two decades, the reported prevalence of synchronous malignancy among HGD patients widely varies from 0–75%. With strict adherence to the Seattle protocol, our model generated a lower estimate of concurrent EAC risk in HGD patients than most published studies, yet it is consistent with the most recent study by Konda et al. when biopsy sensitivity is low [41]. It is also possible that the studies with high estimates of concurrent malignancy were biased because cancer was suspected in these patients indicating esophagectomy.

### Predicted HGD Prevalence with Image-based Screening

High-resolution imaging of BE (a technology still in infancy and not yet widely utilized) may provide a benefit through the early detection and endoscopic resection of small premalignant and malignant lesions. The MSCE-EAC screening model can explore the potential quantitative improvements of screening for neoplasia when diagnosed via optical endomicroscopy compared with a less sensitive biopsy protocol.

To this end, we simulated the results from an optical coherence tomography (OCT) screen in which a positive detection of HGD and/or malignancy occurs if the geometric size of a clone on image is greater than a resolution area threshold, *a*_*OCT*_ (see Methods). Assuming *a*_*OCT*_ = 1mm^2^ and the same assumption for stem cell density *σ* that was used in previous results, the HGD prevalence (excluding incident EAC cases) rose to an expected 27.89% for the BE cohort used in the previous examples (1930 birth year, *t*_*s*_ = 60). Therefore, for the range of probabilities of HGD detection shown in Fig 4, the MSCE-EAC screening model estimated an expected 68.7% to 92.8% increase in HGD detection probability using a sensitive imaging technology for screening rather than biopsy-based screening. This modeling exercise reinforces the conclusion that many neoplastic clones of detectable size are being missed with current biopsy protocol screening endoscopies.

### Predicted EAC Incidence after Treatment

**Fig 6:**
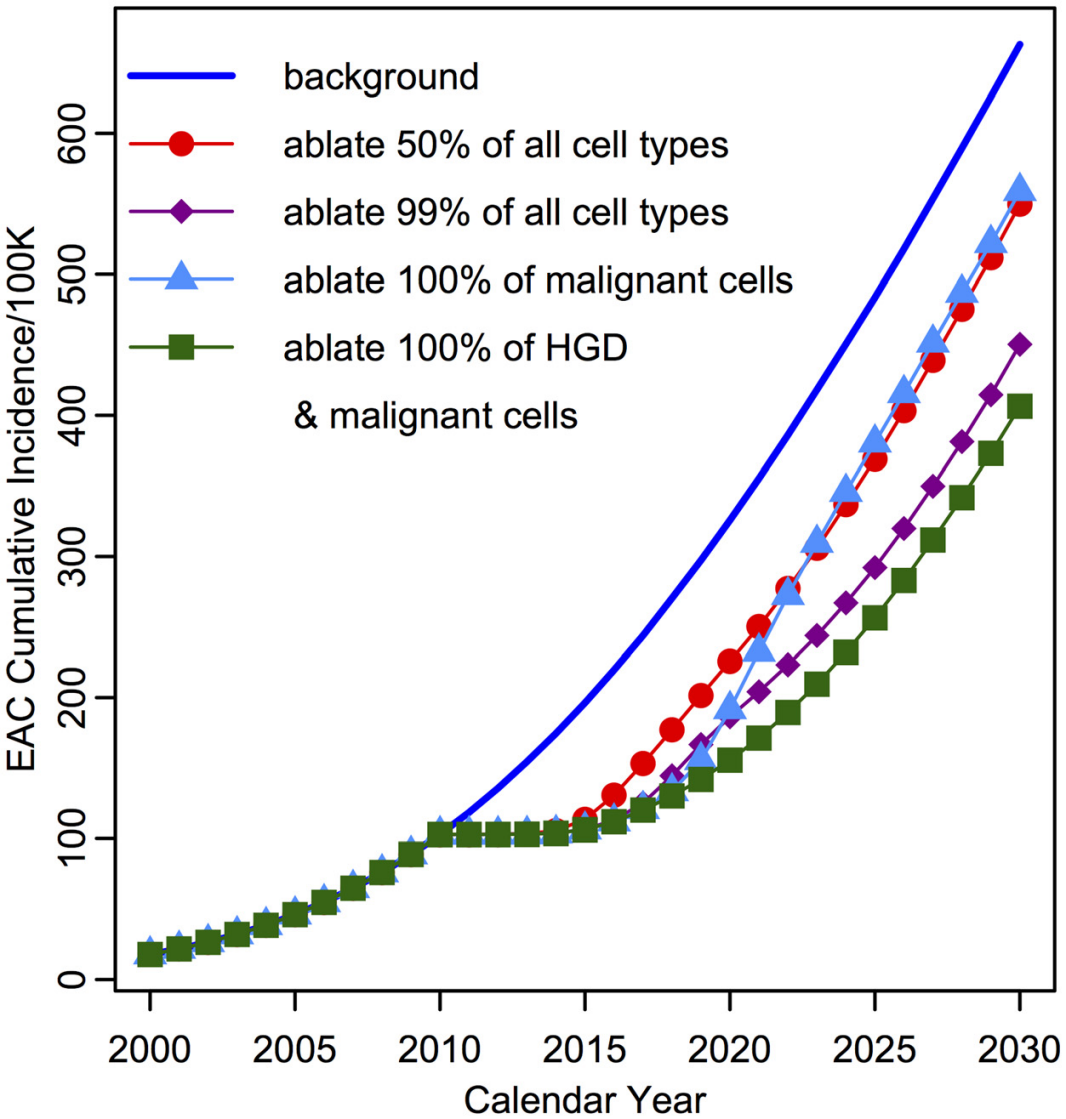
Predicted EAC cumulative age-specific incidence by the MSCE-EAC screening model after RFA treatment of HGD patients at index screen. EAC cumulative age-specific incidence for four different ablation efficiencies of detected HGD patients at screening of 60 year old males in year 2010. Survival for the four cell types following ablation modeled are represented by ablation proportion vector *ω*, with *ω* = {1, 1, 1, 1} (no treatment, blue line), *ω* = {.5, .5, .5, .5} (red, circles), *ω* = {.01, .01, .01, .01} (purple, diamonds), *ω* = {1, 1, 1, 0} (light blue, triangles), *ω* = {1, 1, 0, 0} (green, squares). Scenarios plotted for *σ* = 3300 stem cells/mm^2^ and 60% biopsy sensitivity, resulting in 6% HGD cases, without detected malignancy, in the non EAC population at screening time (100K BE patient simulation size).

As a third example demonstrating the utility of the MSCE-EAC screening model, we computed the projected cumulative hazard Λ_*MSCE*_(*t*) in Eq (3) after a single index screen of BE patients at time *t*_*s*_ = 60, removal of screen-detected EAC patients, and subsequent RFA treatment of HGD positive patients. We explored RFA efficacy under various assumptions about the impact of ablation on cell counts, as specified by the ablation proportion vector *ω* (see Methods). When comparing to the background incidence (in which no screening occurs), we predicted the effect on EAC cumulative incidence based on a range of RFA effectiveness assumptions (See Fig 6). If patients that were positively detected with HGD at index screen (6% with 60% biopsy sensitivity) receive RFA, the MSCE-EAC screening model predicted that by year 2030, expected EAC cumulative incidence will be reduced by 17.1% if 50% of all BE cell types are effectively removed (*ω* = {.5, .5, .5, .5}) and be reduced by 32.1% if 99% of all BE cell types are effectively removed (*ω* = {.01, .01, .01, .01}). To explore the future influence of missed malignancies, the model predicted that if RFA removed all malignancies (*ω* = {1, 1, 1, 0}) but left behind the HGD tissue, then treatment would only moderately reduce future EAC cumulative incidence by an expected 15.7% before 2030. However, removing the HGD tissue as well as preclinical malignancies (*ω* = {1, 1, 0, 0}) during treatment would create a more significant average reduction in EAC cumulative incidence of an expected 38.7%. The model’s predictions of the possible RFA effects on cell populations seem to support the hypothesis that the effectiveness of RFA is determined by its ability to ablate premalignant (dysplastic) tissue.

Interestingly, even the biopsy procedure on all BE patients offers a slight therapeutic effect (EAC cumulative incidence will not return to background) by the mere chance of endoscopically removing, at times, significant amounts of neoplastic tissue in a biopsy specimen, assuming no negative effects from wounding associated with tissue removal. These results are clearly a simplification of a highly variable and complex clinical procedure, representing only a basic example, but the model is poised to incorporate realistic RFA touch-ups throughout surveillance, as it occurs in current practice, to give increasingly realistic projections.

## Discussion

Although few Barrett’s esophagus (BE) patients progress to EAC in their lifetime, the cancer burden is considerable due to generally poor treatment outcomes and survival. EAC contributes approximately 4% to all male cancer deaths in the US [43] with a flattening but still increasing trend in mortality according to recent projections based on Surveillance, Epidemiology, and End Results (SEER) data [12]. Because BE is an actionable EAC precursor with a considerable prevalence of 1–3% in the general population [9, 10] (translating into a large number of individuals) and an annual risk of progressing to EAC of approximately 0.2–0.5% per year [3], optimal surveillance for neoplastic alterations in BE and effective treatment strategies are a major challenge to clinicians given the current lack of evidence-based decision tools. Thus we have developed a detailed multiscale model of EAC to better understand the natural history and impact of screening, intervention, and prevention of EAC.

The mathematical framework of our multistage clonal expansion for EAC (MSCE-EAC) screening model describes the step-wise progression and transformation from normal squamous esophageal tissue to a columnar crypt-structured metaplastic tissue in which clonal expansions of dysplastic and malignant cells can occur. Because the description is fully stochastic, it affords predictions of important clinical endpoints that reflect the intrinsic (inter-individual) heterogeneity in the disease process that explains, at least in part, why some individuals progress to cancer in their life-time while others do not.

In contrast to earlier formulations of the multistage clonal expansion (MSCE) model for EAC [16, 17], which analyzed patterns of EAC incidence in the general population, the present model includes two novel modules for exploration of clinical endpoints before symptomatic detection of EAC. The tissue module explicitly computes the number and sizes of neoplastic clones in a BE patient and quantifies their spatial structure within an idealized crypt-structured BE segment at time of screening. With this patient-specific information, we then employ a screening module to perform a screen *in silico* at a specified screening age. As our BE screening examples demonstrate, this model extension makes it possible to explicitly explore current BE screening efficacy while controlling the operational characteristics of the screening protocol. We show that the detection of high grade dysplasia (HGD) or cancer using the standard (Seattle) biopsy protocol is strongly dependent on the minimum neoplastic tissue fraction needed to be detectable in the biopsy. This sensitivity would be further affected by altering the spacing between biopsy levels and size of the biopsy forceps according to different protocols. Additionally, our MSCE-EAC screening model predicts that over 10% of BE patients screened who receive a diagnosis of HGD with biopsy-based screening also harbor a missed preclinical malignancy with mid-range biopsy sensitivity. We find that the overall efficacy of the biopsy protocol is highly uncertain due to variability in tissue sampling between practitioners and due to considerable uncertainties in the histological assessment of the biopsied tissues.

Our results also suggest that even the best current biopsy protocols may miss between 70%–90% of small HGD lesions that are detectable when using high-resolution optical coherence tomography (OCT) imaging at 1mm resolution. While not yet widely available, high-resolution OCT allows a more complete (wide-field) examination of the BE segment. Our results suggest that OCT could surpass the biopsy-based protocols in efficacy to detect neoplastic lesions. However, because quantitative data with OCT are still lacking, the results remain speculative, but serve to demonstrate the potential gains of OCT screening over the standard biopsy protocol.

Finally, the present framework also allows for the modeling of treatment, such as radio frequency ablation (RFA). Ablation attempts to remove the intestinal metaplasia together with all neoplastic cells. Assuming that ablation simply decimates the number of BE, dysplastic, and malignant crypts by specific fractions, we computed the residual cancer risk of EAC after RFA (see Fig 6). This ‘decimation by fraction’ approach also lends itself to modeling the curative effect of multiple RFA ‘touch-ups’ delivered over a span of time to improve RFA efficacy. From the results derived from simulating an ablative treatment on a population of BE patients found to be positive for HGD during screening, we found that it was crucial to ablate dysplastic and not only preclinical malignant tissue to achieve the most significant impact on future EAC incidence. Although the example given in this study is somewhat simplistic and does not include the random spatial characteristics of the ablation process, the model framework can accommodate more complex assumptions regarding the biological effects of RFA, including random spatial effects of the ablation ‘burn’ and localized presence of intestinal metaplasia hidden beneath the neosquamous tissue after RFA treatment.

In summary, the MSCE-EAC screening model introduced in this paper offers a comprehensive multiscale method to model the neoplastic processes unfolding in BE together with a mechano-spatial modeling of the screening process and treatment. Our results demonstrate the limitations of the standard biopsy-based protocol for the detection of HGD and early cancer due to a highly heterogeneous distribution of dysplastic precursors and malignant foci that can arise in dysplasia. We further demonstrate that these limitations could be overcome by high-resolution OCT imaging which may provide additional biological details and insights into the cancer process, including the growth dynamics of neoplastic clones (in particular their numbers and sizes over time), information that can easily be incorporated into the multiscale description of EAC development and screening presented here.

## Supporting Information

S1 Fig Male symptomatic GERD and BE prevalences in the MSCE-EAC screening model. (Left panel) GERD symptom prevalence *p*_*sGERD*_(*t*). (Right panel) BE prevalence *F*_*BE*_(*t*) for males, assuming *RR* = 5 relative risk for symptomatic GERD patients.
(TIFF)

S2 Fig Female symptomatic GERD and BE prevalences in the MSCE-EAC screening model. (Left panel) GERD symptom prevalence *p*_*sGERD*_(*t*). (Right panel) BE prevalence *F*_*BE*_(*t*) for females, assuming *RR* = 5 relative risk for symptomatic GERD patients.
(TIFF)

S3 Fig Comparison of neoplastic clone growth simulation accuracies using stochastic simulation algorithm (SSA) and *τ*-leaping. Quantile-quantile plots of simulated non-extinct birth-death-mutation processes with *ε* = .0005, stopped at time *t* = 30 years after first *P* cell initiated. Left, SSA sizes vs. geometric distribution sizes. Right, *τ*-leap sizes vs. geometric distribution sizes. Black triangles denote 10th, 50th, and 90th percentiles. Results shown for 100K simulations for each of the three types. Both SSA and *τ*-leap reproduce the theoretical geometric distribution very well.
(TIFF)

S4 Fig Simulated distributions of number and sizes of non-extinct premalignant clones, males. (Left panel) Histogram of number of non-extinct premalignant clones in BE segment at time of screening. (Right panel) Histogram of number of premalignant stem cells in each of the independent premalignant clones accounted for in left panel. Example shown for 100K males with BE, age 60 from the 1930 birth cohort. Median and mean values are depicted by dashed and dotted lines, respectively. Assuming *σ* = 3300 stem cells/mm^2^, the dashed-dotted line on the right graph gives the number of cells in a 1mm^2^ surface area of tissue.
(TIFF)

S5 Fig Simulated distributions of number and sizes of non-extinct malignant clones, males. (Left panel) Histogram of number of non-extinct malignant clones in BE segment at time of screening, each originating from unique premalignant ancestor clone. (Right panel) Histogram of number of malignant stem cells in each of the independent malignant clones accounted for in left panel. Example shown for 100K males with BE, age 60 from the 1930 birth cohort. Median and mean values are depicted by dashed and dotted lines, respectively. Assuming *σ* = 3300 stem cells/mm^2^, the dashed-dotted line on the right graph gives the number of cells in a 1mm^2^ surface area of tissue.
(TIFF)

S6 Fig Simulated distributions of number and sizes of non-extinct premalignant clones, females. (Left panel) Histogram of number of non-extinct premalignant clones in BE segment at time of screening. (Right panel) Histogram of number of premalignant stem cells in each of the independent premalignant clones accounted for in left panel. Example shown for 100K females with BE, age 60 from the 1930 birth cohort. Median and mean values are depicted by dashed and dotted lines, respectively. Assuming *σ* = 3300 stem cells/mm^2^, the dashed-dotted line on the right graph gives the number of cells in a 1mm^2^ surface area of tissue.
(TIFF)

S7 Fig Simulated distributions of number and sizes of non-extinct malignant clones, females. (Left panel) Histogram of number of non-extinct malignant clones in BE segment at time of screening, each originating from unique premalignant ancestor clone. (Right panel) Histogram of number of malignant stem cells in each of the independent malignant clones accounted for in left panel. Example shown for 100K females with BE, age 60 from the 1930 birth cohort. Median and mean values are depicted by dashed and dotted lines, respectively. Assuming *σ* = 3300 stem cells/mm^2^, the dashed-dotted line on the right graph gives the number of cells in a 1mm^2^ surface area of tissue.
(TIFF)

S1 Text Additional mathematical methods and derivations for the MSCE-EAC screening model. This includes derivations and equations for MSCE-EAC model hazard functions, details about parameter estimation, and steps for hybrid algorithm for simulating cell populations.
(PDF)
