# Supplementary figures and images for: A Multiscale Model Evaluates Screening for Neoplasia in Barrett’s Esophagus
Source: PLoS Comput Biol. 2015 May 22;11(5):e1004272. doi: 10.1371/journal.pcbi.1004272 (PMC4441439; doi:10.1371/journal.pcbi.1004272)

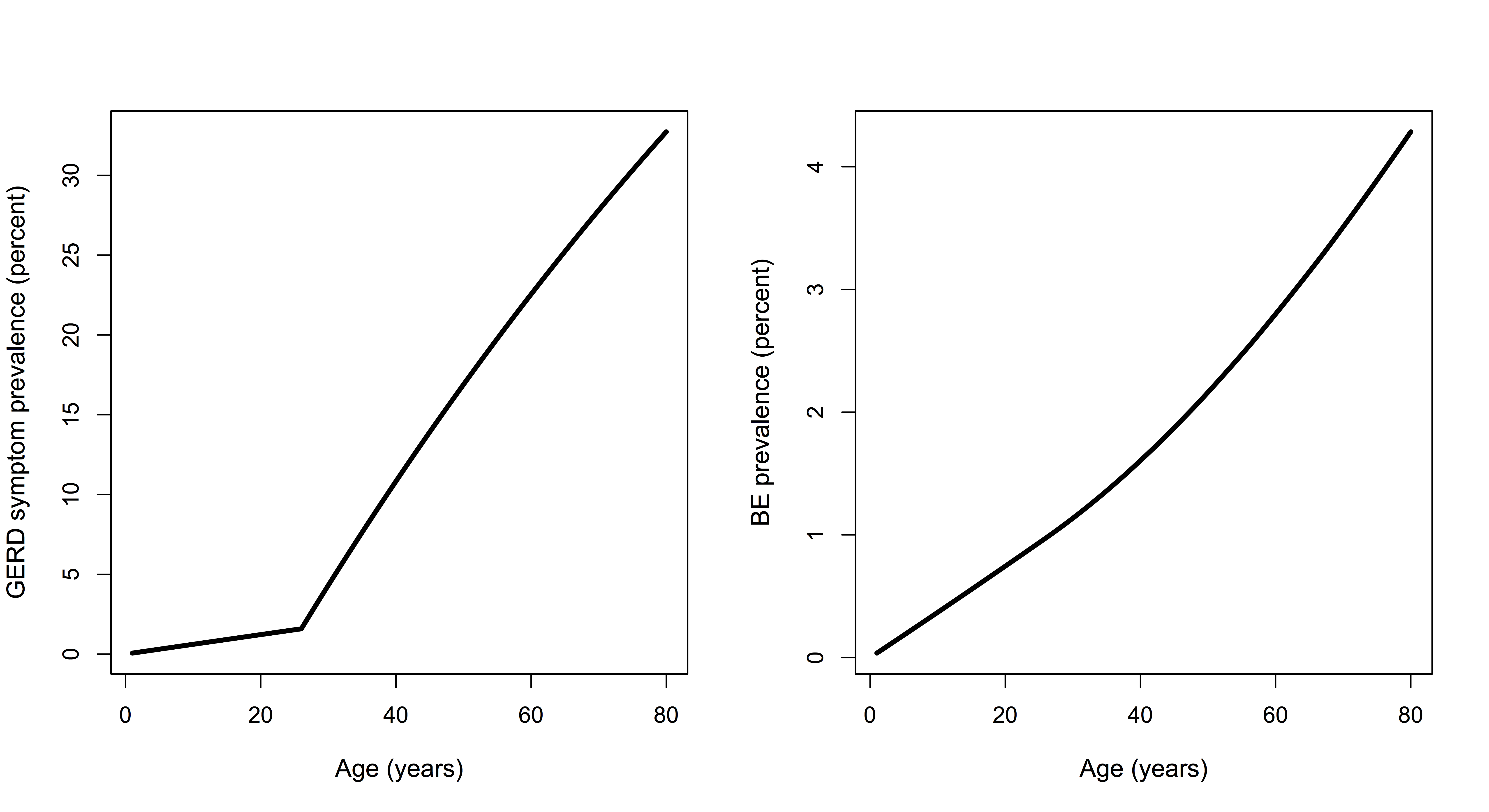

Supplement: S1 Fig — (Left panel) GERD symptom prevalence p sGERD(t). (Right panel) BE prevalence F BE(t) for males, assuming RR = 5 relative risk for symptomatic GERD patients. (TIFF) [file pcbi.1004272.s001.tiff]

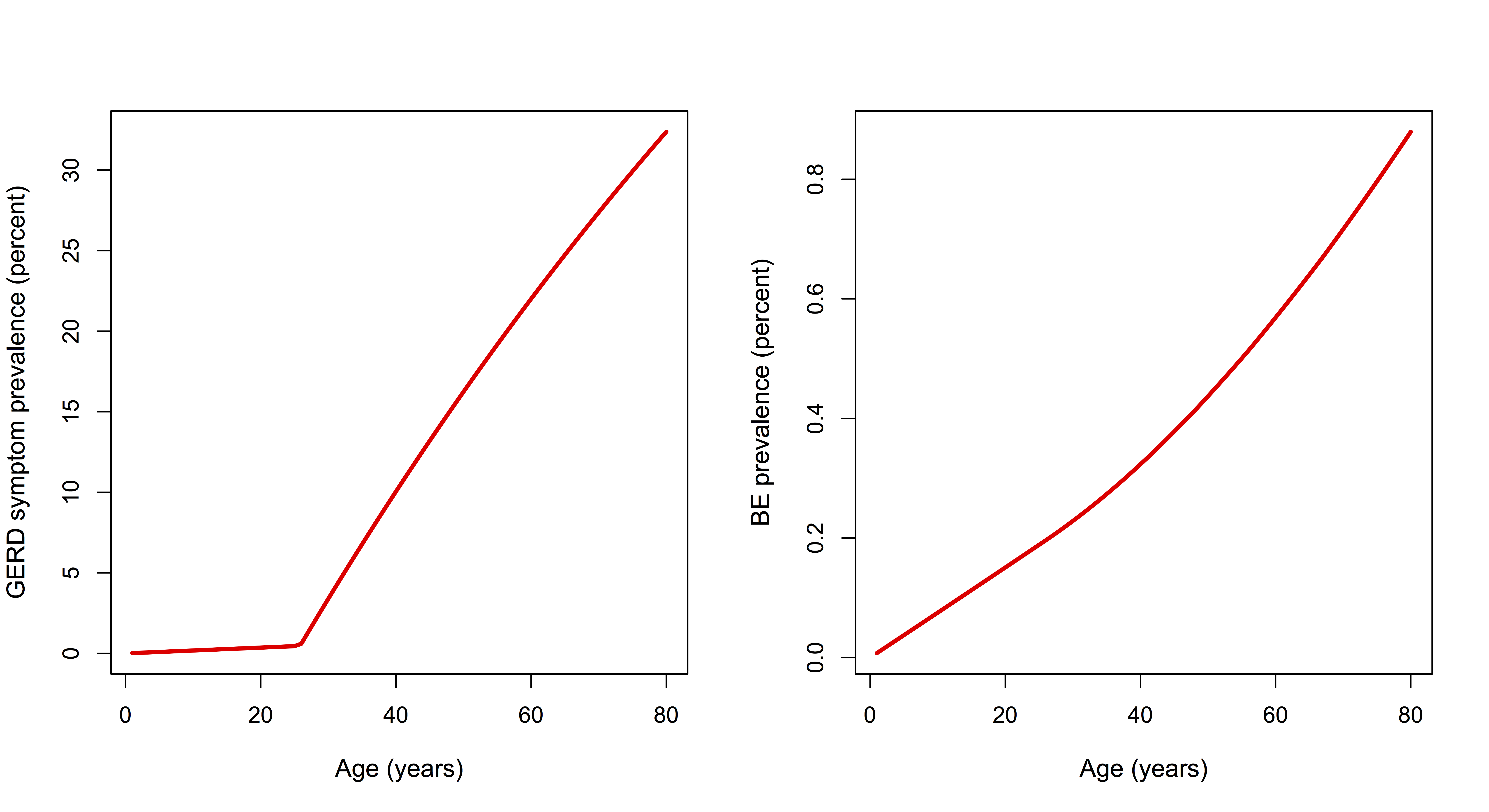

Supplement: S2 Fig — (Left panel) GERD symptom prevalence p sGERD(t). (Right panel) BE prevalence F BE(t) for females, assuming RR = 5 relative risk for symptomatic GERD patients. (TIFF) [file pcbi.1004272.s002.tiff]

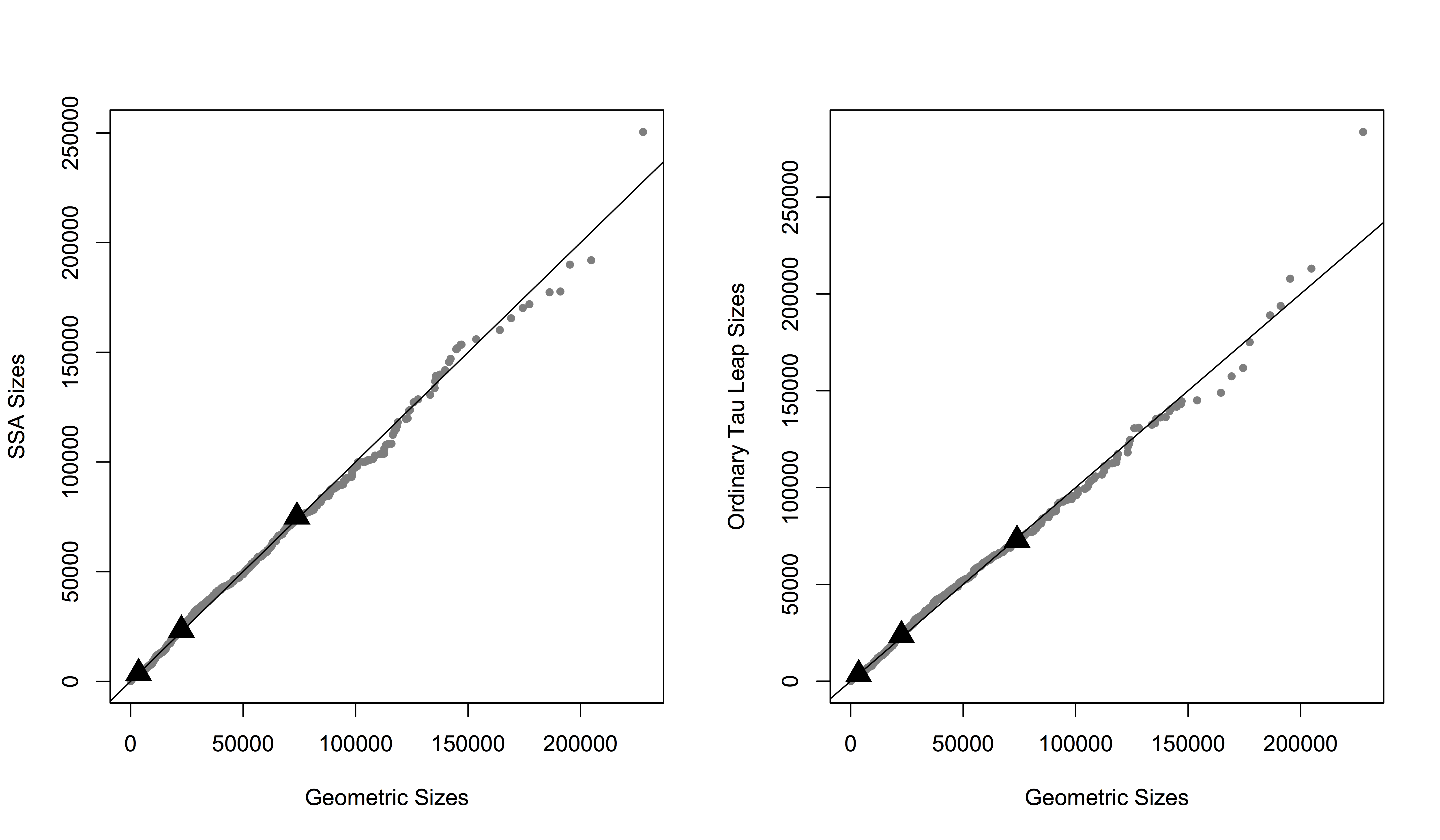

Supplement: S3 Fig — Quantile-quantile plots of simulated non-extinct birth-death-mutation processes with ε = .0005, stopped at time t = 30 years after first P cell initiated. Left, SSA sizes vs. geometric distribution sizes. Right, τ-leap sizes vs. geometric distribution sizes. Black triangles denote 10th, 50th, and 90th percentiles. Results shown for 100K simulations for each of the three types. Both SSA and τ-leap reproduce the theoretical geometric distribution very well. (TIFF) [file pcbi.1004272.s003.tiff]

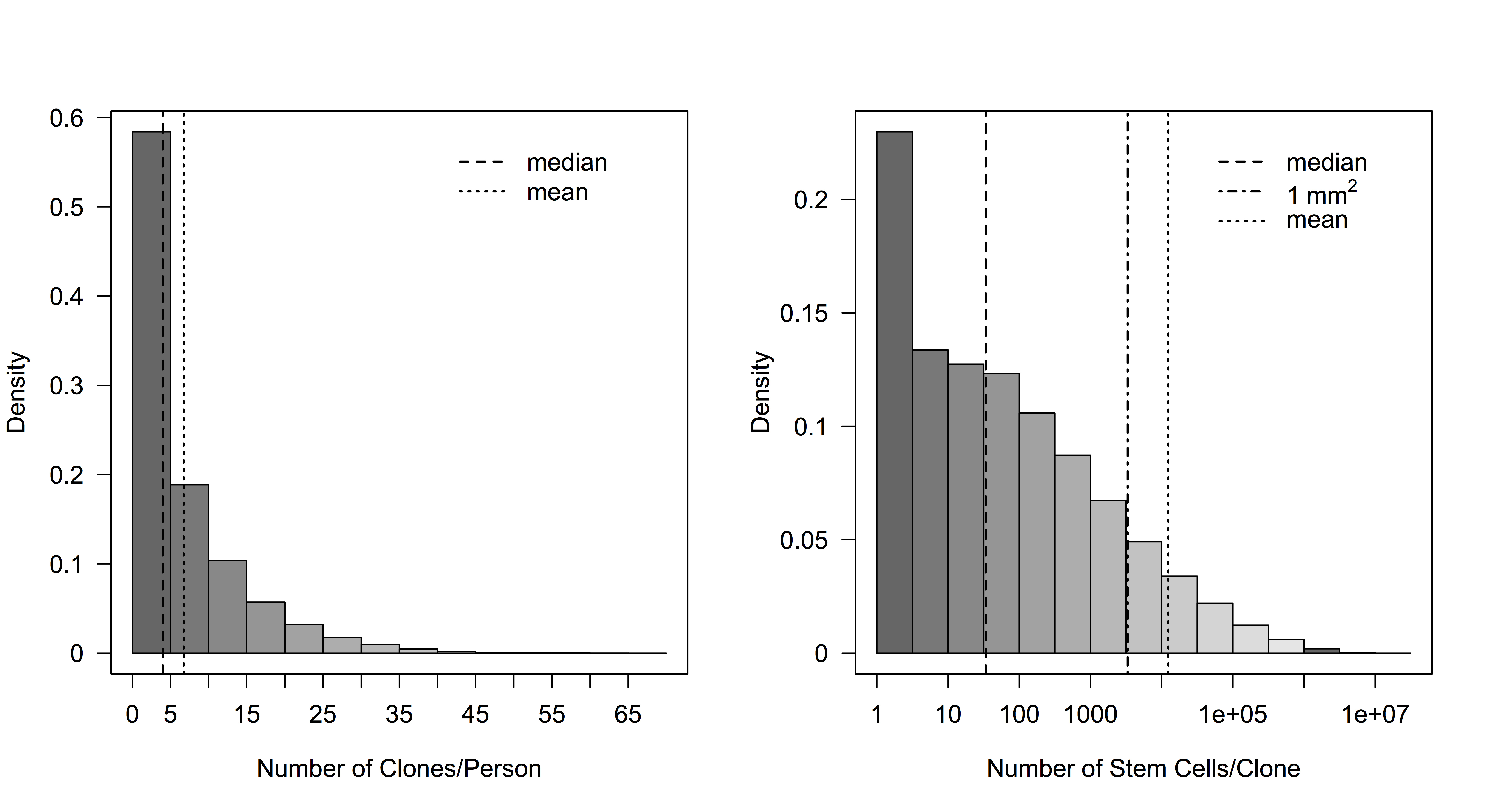

Supplement: S4 Fig — (Left panel) Histogram of number of non-extinct premalignant clones in BE segment at time of screening. (Right panel) Histogram of number of premalignant stem cells in each of the independent premalignant clones accounted for in left panel. Example shown for 100K males with BE, age 60 from the 1930 birth cohort. Median and mean values are depicted by dashed and dotted lines, respectively. Assuming σ = 3300 stem cells/mm2, the dashed-dotted line on the right graph gives the number of cells in a 1mm2 surface area of tissue. (TIFF) [file pcbi.1004272.s004.tiff]

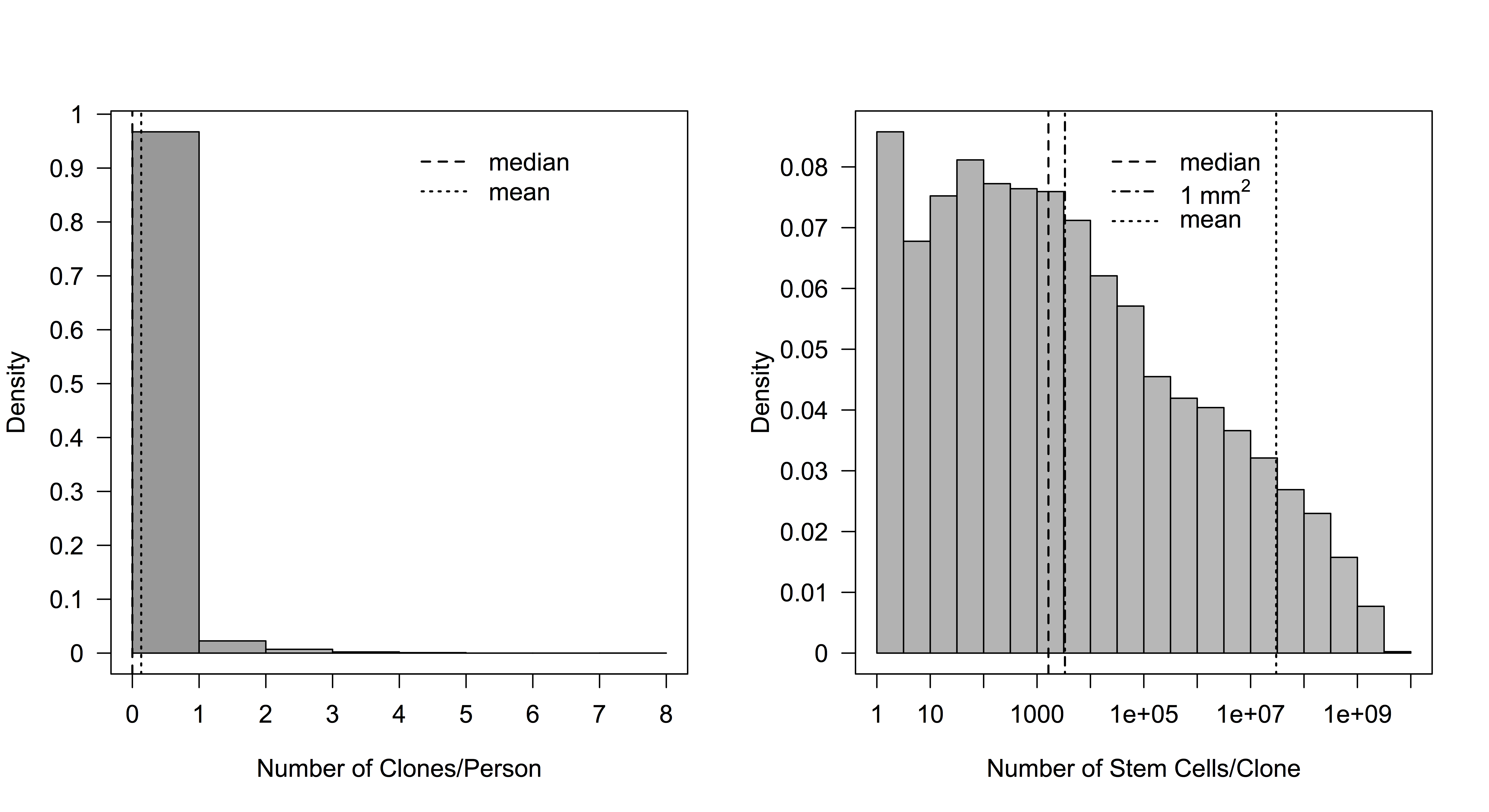

Supplement: S5 Fig — (Left panel) Histogram of number of non-extinct malignant clones in BE segment at time of screening, each originating from unique premalignant ancestor clone. (Right panel) Histogram of number of malignant stem cells in each of the independent malignant clones accounted for in left panel. Example shown for 100K males with BE, age 60 from the 1930 birth cohort. Median and mean values are depicted by dashed and dotted lines, respectively. Assuming σ = 3300 stem cells/mm2, the dashed-dotted line on the right graph gives the number of cells in a 1mm2 surface area of tissue. (TIFF) [file pcbi.1004272.s005.tiff]

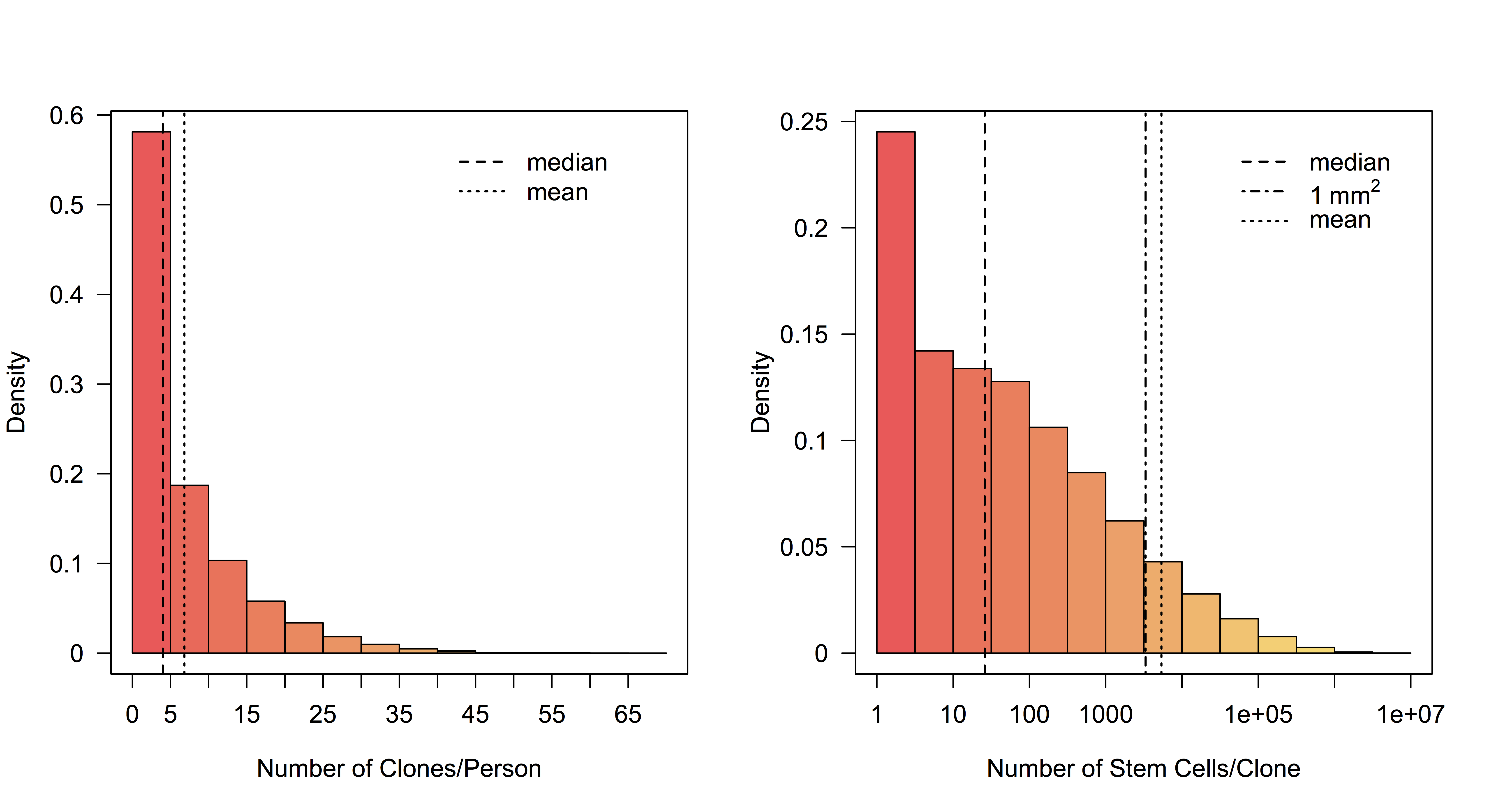

Supplement: S6 Fig — (Left panel) Histogram of number of non-extinct premalignant clones in BE segment at time of screening. (Right panel) Histogram of number of premalignant stem cells in each of the independent premalignant clones accounted for in left panel. Example shown for 100K females with BE, age 60 from the 1930 birth cohort. Median and mean values are depicted by dashed and dotted lines, respectively. Assuming σ = 3300 stem cells/mm2, the dashed-dotted line on the right graph gives the number of cells in a 1mm2 surface area of tissue. (TIFF) [file pcbi.1004272.s006.tiff]

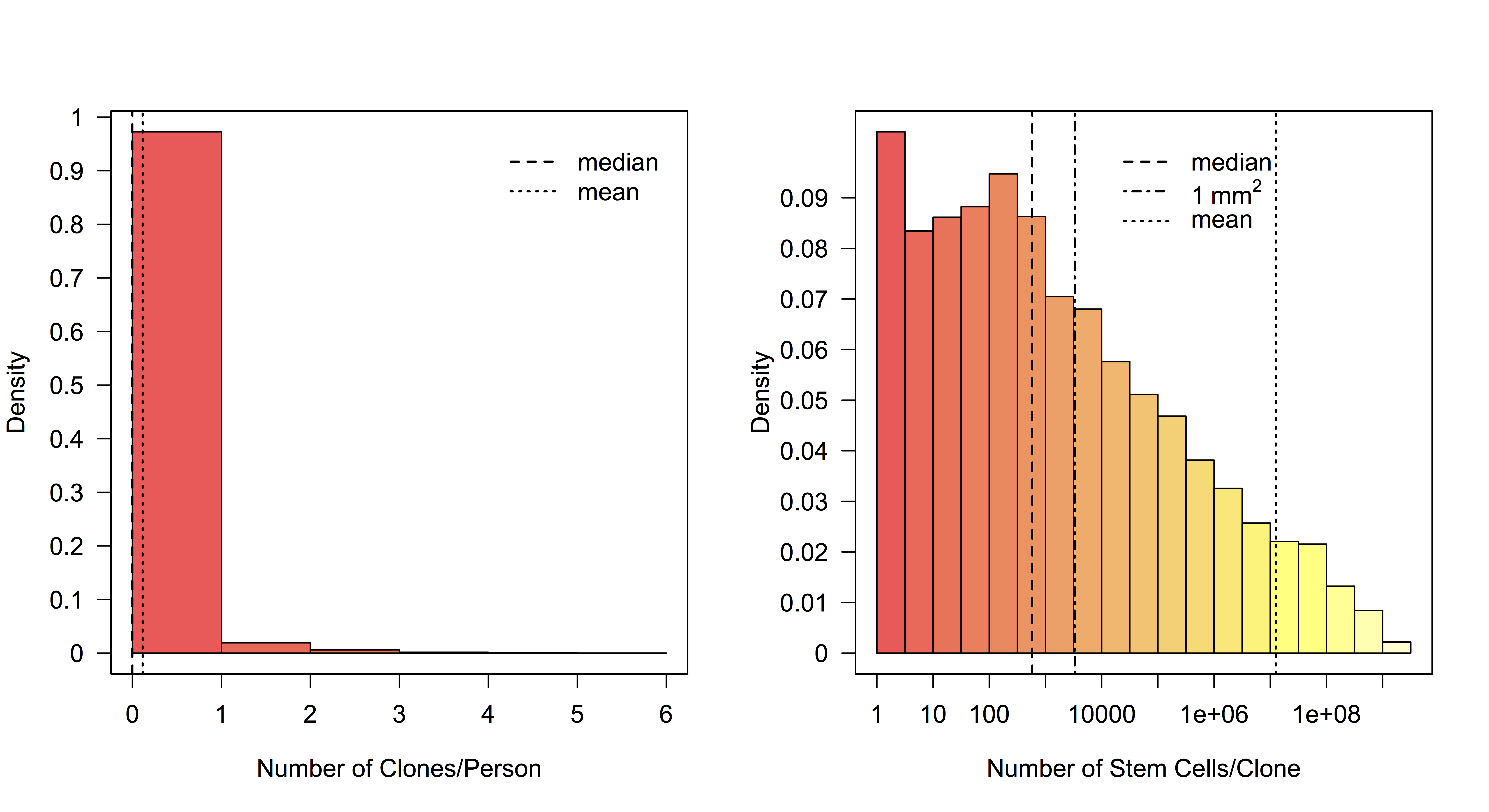

Supplement: S7 Fig — (Left panel) Histogram of number of non-extinct malignant clones in BE segment at time of screening, each originating from unique premalignant ancestor clone. (Right panel) Histogram of number of malignant stem cells in each of the independent malignant clones accounted for in left panel. Example shown for 100K females with BE, age 60 from the 1930 birth cohort. Median and mean values are depicted by dashed and dotted lines, respectively. Assuming σ = 3300 stem cells/mm2, the dashed-dotted line on the right graph gives the number of cells in a 1mm2 surface area of tissue. (TIFF) [file pcbi.1004272.s007.tiff]
