# Supplementary material for: A Multiscale Model Evaluates Screening for Neoplasia in Barrett’s Esophagus
Source: PLoS Comput Biol. 2015 May 22;11(5):e1004272. doi: 10.1371/journal.pcbi.1004272 (PMC4441439; doi:10.1371/journal.pcbi.1004272)
Supplement: S1 Text — This includes derivations and equations for MSCE-EAC model hazard functions, details about parameter estimation, and steps for hybrid algorithm for simulating cell populations. (PDF) [file pcbi.1004272.s008.pdf]

Supporting Information (SI) S1 Text:
A Multiscale Model Evaluates Screening for Neoplasia in Barrett's Esophagus

Mathematical Methods

For each module of the MSCE-EAC screening model described in Methods, we elaborate further on mathematical details in the following sections.

MSCE-EAC cell module: hazard function derivation

We summarize the mathematical development for the multistage clonal expansion for EAC (MSCE-EAC) cellular model (see Fig. 2 in Main Text) and first introduce the notation for the following random variables of the multi-type branching process

$$\begin{aligned}
BE(t) &= \text{Bernoulli random variable for BE conversion by time } t\\
X(t) &= \text{number of BE stem cells in a tissue at time } t\\
P^{*}(t) &= \text{number of pre-initiated cells at time } t\\
P(t) &= \text{number of premalignant (initiated) cells at time } t\\
M(t) &= \text{number of malignant (preclinical) cells (prior to detection) at time } t\\
C(t) &= \text{number of cancer cells (after detection) at time } t\\
D(t) &= \text{Bernoulli random variable for clinical detection by time } t
\end{aligned}$$

Let us consider the probability generating function (pgf) Ψ for the entire process starting at $\tau = 0$, ie. when an individual is born

$$\Psi(y_{BE},y_1,y_2,y_3,z;t) = \sum_{i,j,k,l,n} y_{BE}^{i} y_1^{j} y_2^{k} y_3^{l} z^{n} P(i,j,k,l,n;t),$$

$$P(i,j,k,l,n;t) = \Pr[BE(t)=i, P^{*}(t)=j, P(t)=k, M(t)=l, D(t)=n \,|\, BE(0)=0, P^{*}(0)=0, P(0)=0, M(0)=0, D(0)=0]$$

where, explicitly, $i, n = \{0, 1\}$ and $BE(t), D(t)$ are the following indicator functions corresponding to BE conversion and EAC clinical detection, respectively

$$BE(t) = \begin{cases} 0 & \text{if no BE has not developed by time } t \\ 1 & \text{if BE conversion has taken place by time } t \end{cases}$$

$$D(t) = \begin{cases} 0 & \text{if no cancer detected clinically by time } t \\ 1 & \text{if a malignant cell is detected by time } t. \text{ ie } C(\tau) > 0 \text{ for some } \tau \leq t \end{cases}$$

The Chapman-Kolmogorov equations governing the transition probabilities for this multistage process include contributions from the initial Armitage-Doll type transition to BE, the two Poisson transitions to initiation, and the two birth-death-migration processes, all of which have been derived previously [1–3]. We begin with the forward Kolmogorov differential equation for the entire process $\Psi(y_{BE}, y_1, y_2, y_3, z; \tau = 0, t)$, given by

$$\begin{aligned}\frac{\partial\Psi(y_{BE},y_1,y_2,y_3,z;t)}{\partial t} &= \nu(t)y_{BE} - \nu(t)\Psi - \mu_0 X(1-y_1)y_{BE}\frac{\partial\Psi}{\partial y_{BE}} - \mu_1(1-y_2)y_1\frac{\partial\Psi}{\partial y_1} \\ &+ [\beta_P + \alpha_P y_2^2 - \{\beta_P + \alpha_P + \mu_2(1-y_3)\}y_2]\frac{\partial\Psi}{\partial y_2} \\ &+ [\beta_M + \alpha_M y_3^2 - \{\beta_M + \alpha_M + \rho(1-z)\}y_3]\frac{\partial\Psi}{\partial y_3}\end{aligned} \tag{1}$$

where we have suppressed the dependence on $(y_{BE}, y_1, y_2, y_3, z; t)$ in Ψ for convenience. This high-dimensional PDE poses numerical issues but we shall show a more amenable method for solving the generating function using the Kolmogorov backward equations.

Backward Kolmogorov equations and the MSCE-EAC hazard, h_{MSCE}

Beginning with an active BE segment (BE), a single pre-initiated (P^*), premalignant (P), or malignant (M) cell at time τ only, we define the following generating functions Φ_{BE},Φ_{P^*}, Φ_P, or Φ_M, respectively,

$$\begin{aligned}\Phi_M(y_3,z;\tau,t) &= E[y_3^{M(t)}z^{D(t)}|M(\tau)=1,D(\tau)=0] \\ &= \sum_{k,l} y_3^k z^l \Pr[M(t)=k,D(t)=l|M(\tau)=1,D(\tau)=0]\end{aligned} \tag{2}$$

$$\begin{aligned}\Phi_P(y_2,y_3,z;\tau,t) &= E[y_2^{P(t)}y_3^{M(t)}z^{D(t)}|P(\tau)=1,M(\tau)=0,D(\tau)=0] \\ &= \sum_{j,k,l} y_2^j y_3^k z^l \Pr[P(t)=j,M(t)=k,D(t)=l|P(\tau)=1,M(\tau)=0,D(\tau)=0]\end{aligned} \tag{3}$$

$$\begin{aligned}\Phi_{P^*}(y_1,y_2,y_3,z;\tau,t) &= E[y_1^{P^*(t)}y_2^{P(t)}y_3^{M(t)}z^{D(t)}|P^*(\tau)=1,P(\tau)=0,M(\tau)=0,D(\tau)=0] \\ &= \sum_{i,j,k,l} y_1^i y_2^j y_3^k z^l \Pr[P^*(t)=i,P(t)=j,M(t)=k,D(t)=l|P^*(\tau)=1,P(\tau)=0,M(\tau)=0,D(\tau)=0]\end{aligned} \tag{4}$$

$$\Phi_{BE}(y_{BE},y_1,y_2,y_3,z;\tau,t) = E[y_{BE}^{BE(t)}y_1^{P^*(t)}y_2^{P(t)}y_3^{M(t)}z^{D(t)}|BE(\tau)=1,P^*(\tau)=0,P(\tau)=0,M(\tau)=0,D(\tau)=0] \tag{5}$$

$$= \sum_{i,j,k,l,n} y_{BE}^i y_1^j y_2^j y_3^l z^n \Pr[BE(t)=i,P^*(t)=j,P(t)=k,M(t)=l,D(t)=n|BE(\tau)=1,P^*(\tau)=0,P(\tau)=0,M(\tau)=0,D(\tau)=0]$$

The generating functions satisfy the following Kolmogorov backward equations

$$\begin{aligned}\frac{\partial\Phi_M(y_3,z;\tau,t)}{\partial\tau} &= -\alpha_M\Phi_M^2(y_3,z;\tau,t) - \beta_M \\ &- z\rho\Phi_M(y_3,z;\tau,t) + [\alpha_M + \beta_M + \rho]\Phi_M(y_3,z;\tau,t)\end{aligned} \tag{6}$$

$$\begin{aligned}\frac{\partial\Phi_P(y_2,y_3,z;\tau,t)}{\partial\tau} &= -\alpha_P\Phi_P^2(y_2,y_3,z;\tau,t) - \beta_P \\ &+ [\alpha_P + \beta_P + \mu_2]\Phi_P(y_2,y_3,z;\tau,t) - \mu_2\Phi_P(y_2,y_3,z;\tau,t)\Phi_M(y_3,z;\tau,t)\end{aligned} \tag{7}$$

$$\frac{\partial\Phi_{P^*}(y_1,y_2,y_3,z;\tau,t)}{\partial\tau} = -\mu_1\Phi_{P^*}(y_1,y_2,y_3,z;\tau,t)[\Phi_P(y_2,y_3,z;\tau,t) - 1] \tag{8}$$

$$\frac{\partial\Phi_{BE}(y_{BE},y_1,y_2,y_3,z;\tau,t)}{\partial\tau} = -\mu_0 X\Phi_{BE}(y_{BE},y_1,y_2,y_3,z;\tau,t)[\Phi_{P^*}(y_1,y_2,y_3,z;\tau,t) - 1] \tag{9}$$

$$\frac{\partial\Psi(y_{BE},y_1,y_2,y_3,z;\tau,t)}{\partial\tau} = \nu(\tau)[\Psi(y_{BE},y_1,y_2,y_3,z;\tau,t) - \Phi_{BE}(y_{BE},y_1,y_2,y_3,z;\tau,t)] \tag{10}$$

To connect the cellular level description to the population level, we first solve for the overall survival function (for EAC cancer detection), starting at time 0, which in our notation is

$$S_{MSCE}(t) = 1 - P_{MSCE}(t) = \Pr[D(t) = 0 | BE(0) = 0, P^*(0) = 0, P(0) = 0, M(0) = 0, D(0) = 0]$$
$$= \Psi(1, 1, 1, 1, 0; 0, t)$$

where $P_{MSCE}(t)$ is the probability of a cancer detection at time t,

$$P_{MSCE}(t) = \Pr[D(t) = 1 | BE(0) = 0, P^*(0) = 0, P(0) = 0, M(0) = 0, D(0) = 0]$$

We will here denote $\Phi_M(1, 0; \tau, t) \equiv \Phi_M(\tau, t)$, $\Phi_P(1, 1, 0; \tau, t) \equiv \Phi_P(\tau, t)$, $\Phi_{P*}(1, 1, 1, 0; \tau, t) \equiv \Phi_{P*}(\tau, t)$, $\Phi_{BE}(1, 1, 1, 1, 0; \tau, t) \equiv \Phi_{BE}(\tau, t)$, and $\Psi(1, 1, 1, 1, 0; \tau, t) \equiv \Psi(\tau, t)$. A dot designates a first derivative with respect to t. The hazard function, i.e., the rate at which cancer is detected in individuals who have not been diagnosed before, is given by

$$h_{MSCE}(t) = -\frac{\dot{S}_{MSCE}(t)}{S_{MSCE}(t)} = -\frac{\dot{\Psi}(0, t)}{\Psi(0, t)} = -\frac{d}{dt}\ln[\Psi(0, t)] \quad (11)$$

For fixed t, this boundary value system of coupled PDEs can be converted into an initial value problem (IVP) with the change of variables $u = t - \tau$, where u is the "running" time. This redefinition and equations hereafter follow the method used by Crump et al. [4]. Define the following variables for the new IVP: $Y_1(u, t) = \Phi_M(\tau, t), Y_2(u, t) = \dot{\Phi}_M(\tau, t), Y_3(u, t) = \Phi_P(\tau, t), Y_4(u, t) = \dot{\Phi}_P(\tau, t), Y_5(u, t) = \Phi_{P*}(\tau, t), Y_6(u, t) = \dot{\Phi}_{P*}(\tau, t), Y_7(u, t) = \Phi_{BE}(\tau, t), Y_8(u, t) = \dot{\Phi}_{BE}(\tau, t), Y_9(u, t) = \Psi(\tau, t), Y_{10}(u, t) = \dot{\Psi}(\tau, t)$ with corresponding initial conditions $Y_1(0, t) = Y_3(0, t) = Y_5(0, t) = Y_7(0, t) = Y_9(0, t) = 1, Y_4(0, t) = Y_6(0, t) = Y_8(0, t) = Y_{10}(0, t) = 0$, and $Y_2(0, t) = -\rho$. Then the equations to solve for our IVP are the following

$$\frac{dY_1(u,t)}{du} = \beta_M - (\alpha_M + \beta_M + \rho)Y_1(u,t) + \alpha_M Y_1^2(u,t) \quad (12)$$

$$\frac{dY_2(u,t)}{du} = 2\alpha_M Y_1(u,t)Y_2(u,t) - (\alpha_M + \beta_M + \rho)Y_2(u,t) \quad (13)$$

$$\frac{dY_3(u,t)}{du} = \beta_P + \mu_2 Y_1(u,t)Y_3(u,t) - (\alpha_P + \beta_P + \mu_2)Y_3(u,t) + \alpha_P Y_3^2(u,t) \quad (14)$$

$$\frac{dY_4(u,t)}{du} = 2\alpha_P Y_3(u,t)Y_4(u,t) + \mu_2(Y_4(u,t)Y_1(u,t) + Y_3(u,t)Y_2(u,t)) - (\alpha_P + \beta_P + \mu_2)Y_4(u,t) \quad (15)$$

$$\frac{dY_5(u,t)}{du} = \mu_1 Y_5(u,t)(Y_3(u,t) - 1) \quad (16)$$

$$\frac{dY_6(u,t)}{du} = \mu_1(Y_6(u,t)Y_3(u,t) - Y_6(u,t) + Y_5(u,t)Y_4(u,t)) \quad (17)$$

$$\frac{dY_7(u,t)}{du} = \mu_0 X Y_7(u,t)(Y_5(u,t) - 1) \quad (18)$$

$$\frac{dY_8(u,t)}{du} = \mu_0 X(Y_8(u,t)Y_5(u,t) - Y_8(u,t) + Y_7(u,t)Y_6(u,t)) \quad (19)$$

$$\frac{dY_9(u,t)}{du} = \nu(u)(Y_7(u,t) - Y_9(u,t)) \quad (20)$$

$$\frac{dY_{10}(u,t)}{du} = \nu(u)(Y_{10}(u,t) - Y_8(u,t)) \quad (21)$$

These 10 coupled ODEs can be solved numerically to obtain

$$h_{MSCE}(t) = -Y_{10}(t,t)/Y_9(t,t) \quad \text{and} \quad S_{MSCE}(t) = Y_9(t,t). \tag{22}$$

Non-spatial parameter estimation with birth cohort trends

Here we describe the methods and values used for the non-spatial parameter estimates used in [5]. The fitted non-spatial parameters from Kong et al. are provided in Table 1 and used for the Results in the Main Text. We modeled gastroesophageal reflux disease (GERD) symptom prevalence at age t, $p_{sGERD}(t)$, based on data from Ruigomez, et al. [6] for incidence (by 2-year age intervals) of GERD symptoms (that occur weekly or more frequently) among children (n=1700), and another study by Ruigomez, et al. [7] on incidence of weekly GERD symptoms among adults (n=1996) with data provided in 10 year intervals. We used maximum likelihood methods to fit parameters for a GERD prevalence model separately for males and females, using a transition rate to GERD prevalence based on the GERD incidence data and estimating a back-transition rate (representing recovery from GERD) to fit an assumed 20% target rate for age-adjusted GERD prevalence between ages 40-85 [8]. We then found that we could achieve excellent fits to these data by using simplified, gender-specific models with three parameters representing a (slower) transition rate among children, a transition age, and an adult rate for acquiring weekly GERD symptoms. BE prevalence, $F_{BE}(t)$, can be estimated, via parameter ν_0, and fixing a value for relative risk $RR = 5$ (based on results from meta-analyses for BE segments greater than 3 cm in length [9]), using the model for GERD prevalence as described in the Main Text with the following BE conversion rate

$$\nu(t) = \nu_0 \left((1 - p_{sGERD}(t)) + RR \cdot p_{sGERD}(t)\right) \tag{23}$$

$$\Rightarrow F_{BE}(t) = \Pr[T_{BE} \le t] = 1 - e^{-\int_0^t \nu(s)\,\mathrm{d}s} \tag{24}$$

See S1-S2 Figures for values $p_{sGERD}(t)$ and $F_{BE}(t)$ used for males and females, respectively.

In Kong et al., we employed an Age-Cohort (AC) model, which models the birth cohort effect as a sigmoid function on both premalignant and malignant proliferation and cell division rates $g_P, \alpha_P, g_M, \alpha_M$. Assumptions must be made for values of the background cell division rates, $\alpha_{M,0}$ and $\alpha_{P,0}$. The model estimates values for $g_{P,0}, g_1, g_2, g_3$ (reference year), where t_b = birth cohort year, with the following functional forms

$$g_P = g_{P,0}\left(g_1 + \frac{2}{1 + e^{(-g_2(t_b - g_3))}}\right), \;\; g_M = g_{M,0}\left(g_1 + \frac{2}{1 + e^{(-g_2(t_b - g_3))}}\right)$$

$$\alpha_P = \alpha_{P,0}\left(g_1 + \frac{2}{1 + e^{(-g_2(t_b - g_3))}}\right), \;\; \alpha_M = \alpha_{M,0}\left(g_1 + \frac{2}{1 + e^{(-g_2(t_b - g_3))}}\right)$$

$$g_P = \alpha_P - \beta_P - \mu_2, \;\; g_M = \alpha_M - \beta_M - \rho$$

We assumed that all other biological parameters, except for the BE conversion rate $\nu(t)$ that depends on age, are constant.

All cellular kinetic model parameters were estimated using a maximum likelihood method to obtain best fits of the hazard function given in Eq. (22) to SEER incidence data [5, 10]. We obtained the 95% confidence intervals for these estimates using a Markov-Chain Monte Carlo (MCMC) method, in which all runs were started with the non-spatial parameters set at (or

near) their respective maximum likelihood estimates (MLEs) and appeared to converge rapidly after a short 1000 cycle burn-in period. However, not all of the MSCE-EAC model non-spatial parameters are identifiable from incidence data - some parameters must be fixed initially in order to achieve parameter identifiability (see Heidenreich et al. [11]). For example, the hazard function yields estimates for the product of the rates for the first two rate-limiting events, μ_0 and μ_1 and the average number of non-neoplastic BE stem cells X. Thus we set $\mu_0 = \mu_1$ and fix X when reporting and utilizing estimates. We also assumed $\alpha_{P,0} = 10$ per cell/year, and $\alpha_{M,0} = 150$ per cell/year to attain identifiability of the premalignant and malignant growth parameters. The maximum likelihood values were estimated using the Davidon-Fletcher-Powell gradient search method, available in the R software Bhat package, written by Dr. Georg Luebeck.

We compared multiple models by fixing $g_{M,0}$ and detection rate ρ to different values in order to achieve reasonable mean sojourn times and tumor doubling times that are in line with clinical data. From this previous analysis [5], we estimated the EAC clinical detection rate $\rho = 10^{-9}$ per cell/year, malignant cell proliferation rate $g_{M,0} = 0.75$ per cell/year, and the number of stem cells in an average 5 cm BE segment $X = 10^6$ to accompany the estimates provided in Table 1.

MSCE-EAC tissue module: hybrid simulation algorithm

The following steps describe the MSCE-EAC tissue module simulation for a single individual's MSCE-EAC multi-type branching process realization from birth until time of screening t_s

Step 1: Simulate BE onset time

For each individual, generate an age of BE onset, T_{BE} using the inverse cumulative distribution technique with Eq. (24). Because the incidence rate of BE in the general population is very small, most random realizations of this onset time will be longer than the human lifespan. The simulation continues only for those individuals who have a T_{BE} onset time within the age range of interest (possibly before a set screening age t_s).

Step 2: Generate BE segment length

Generate the size of a patient's BE segment (measured clinically as the length from gastroesophageal (GE) junction to the top of the longest "tongue" of metaplastic tissue) as a random deviate from a length distribution based on clinical studies [12–17]. This length will be translated into a number of BE stem cells, X, which depends on a spatial model parameter σ for the density of stem cells per mm^2. The MSCE-EAC model assumes that BE stem cells are under tight homeostatic control with zero net growth of the non-dysplastic BE stem cell population. Mutations and clonal populations occurring during the simulation grow within this fixed BE segment.

Step 3: Generate pre-initiated stem cells P^*

Any of the BE stem cells, total of X, may undergo a Poisson rate-limiting mutation with rate μ_0 during an asymmetric division to produce a BE daughter stem cell and a pre-initiated P^* cell. A P^* cell may arise through inactivation of a single tumor suppressor allele. To model this process up until time of screening t_s, generate a number N_{P*} of P^* pre-initiation events from a Poisson distribution with mean $\mu_0 \cdot X \cdot (t_s - T_{BE})$, each of which occur at a uniform time τ between T_{BE} and t_s, and save in vector $\vec{\tau}_1$.

Step 4: Generate initiated stem cells P and premalignant clones

Similarly, each P^* cell may undergo a second Poisson rate-limiting mutation with rate μ_1 during

an asymmetric division to produce one P^* daughter stem cell and one initiated P cell. A P cell may be a cell with a tumor suppressor gene that has both alleles inactivated, which will allow it to undergo clonal expansion as an independent birth-death-mutation process. Again, for each P^* cell born at time $\tau_{1i}, i = 1, .., N_{P*}$, generate the number N_P of initiated P progenitor cells from a Poisson distribution with mean $\mu_1 \cdot (t_s - \tau_{1i})$, each of which occurs at a uniformly distributed time between τ_{1i} and t_s, and save in vector $\vec{\tau}_2$.

For each P cell initiation, begin a simulation of the ensuing birth-death-mutation (b-d-m) process to follow the number and times of symmetric divisions, death or differentiation, and malignant transformations that occur in each premalignant clone.

Step 5: Generate preclinical cancer cells M, malignant clones, and clinical EAC detection

During simulation of premalignant clones, malignant transformations may occur within a particular clone, modeled as asymmetric divisions of a P cell with rate μ_2. For each malignant progenitor M cell born at time τ_3, begin an independent birth-death-detection process that is represented by an analytical solution to the corresponding Kolmogorov equation for the generating function as derived in Eq. (3.5) of Jeon et al. [18]. Thus, the hybrid simulation makes use of previous theoretical results for an analytical distribution to avoid further simulation. We are first interested in knowing whether a malignant clone born at time τ_3 leads to a clinical EAC by time t_s. To generate this potential outcome, we use the 1-stage *survival function* S_M,

$$S_M(u) \equiv \Pr[D(t_s) = 0 | M(\tau_3) = 1] \tag{25}$$

where $u = t_s - \tau_3$, $D(t_s)$ is the random variable for clinical detection by time t_s, and $M(\tau_3)$ is the random number of malignant (preclinical) cells at time τ_3 in a malignant clone. Letting $p = 1 - S_M(t_s - \tau_3)$ represent the probability of cancer detection of a particular malignant clone born at time τ_3, draw a Bernoulli random variable with probability p to decide if this clone will be detected as a clinical EAC by time t_s. Draw Bernoulli deviates from detection probability p for each malignant clone generated in a patient, and repeat for every patient in the simulated population to obtain the EAC detection prevalence by time t_s.

For patients in whom a malignant clone born at time τ_3 does not result in clinical EAC by time t_s, use an analytical distribution to generate the size of the malignant clone present at time t_s, conditional on no EAC detection. Jeon et al. [18] derived this conditional size distribution for a birth-death-detection process, which is a shifted geometric distribution, described in more detail forthcoming.

This step completes the MSCE-EAC hybrid simulation of an individual from birth until time (age) t_s which can be repeated to generate (synthetic) data for a sample population. In summary, for those individuals who are found to have BE by time of screening, each patient has a specific X number of BE stem cells, P^* number of pre-initiated cells, a number of non-extinct P clones with respective sizes, a number of non-extinct M clones with respective sizes and information about the parental P clones from which the M clones originated, and lastly whether the patient is a prevalent, clinical EAC case by time t_s. We tested the full MSCE-EAC simulation accuracy through comparison of the number of EAC cases simulated with those predicted by the analytical MSCE-EAC cumulative hazard function.

Implementation of SSA and τ-leap method for P clones

As mentioned in Step 3 of the MSCE-EAC algorithm above, initiated premalignant clones undergo independent birth-death-mutation (b-d-m) processes that we simulate to track cell

count and times of malignant transformations. The stochastic simulation algorithm (SSA) is a mathematically exact method to follow each event that occurs during a realization of the continuous time Markov chain beginning with a single cell. Considering an individual premalignant clone of size X_t at time t, we define the intensity function vector $r(X_t) = (\beta_P X_t, \mu_2 X_t, \alpha_P X_t)$ for death/differentiation, malignant transformation, and birth of new P stem cell, where, over a short period of time s, we expect $r_j(X_t)s + o(s)$ events of type j to occur. Due to the Markovian property of the process, we wait an exponential length of time until the next event occurs with intensity $r_0(X_t) = \sum_{j=1}^{3} r_j(X_t) = X_t(\beta_P + \mu_2 + \alpha_P)$. Once an exponential time to next event is chosen, we jump to the neighboring state $X_t + v_j$ with probability $r_j(X_t)/r_0(X_t)$, where v_j is the jth component of the state change vector $v = (-1, 0, 1)$ for the b-d-m process. Fortunately, in the case of the P clone process with constant rates, the probabilities $r_j(X_t)/r_0(X_t)$ are constant with respect to the current state X_t so we may generate a number K of events of the three types with probabilities $\left(\frac{\beta_P}{\beta_P+\mu_2+\alpha_P}, \frac{\mu_2}{\beta_P+\mu_2+\alpha_P}, \frac{\alpha_P}{\beta_P+\mu_2+\alpha_P}\right)$ and cumulatively sum each $X_t + v^j$ step for the K chosen events to create a state vector N. Then we generate the K exponential waiting times of the process at once from an exponential with mean $\lambda_t = N(\beta_P + \mu_2 + \alpha_P)$ and cumulatively sum these to arrive at a new later time $t_2 > t$.

The SSA works very well when cell count of the P clone is small and the event intensities $r(X_t)$ are fluctuating quickly. In particular, our simulation benefits to use the SSA for the beginning of a P clone's growth from a single cell, when the probability of extinction is high (β_P is only slightly smaller than α_P) and most clones are eliminated after a small number K of initial events. However, the SSA can become excruciatingly slow when a P clone becomes very large, i.e. contains a large number of stem cells. Therefore, rather than simulating every event choice and time, we can employ an accelerated but approximate procedure called the τ-leap method, first introduced by Gillespie and others [19–21]. The goal of this procedure is to advance the cell count by a preselected time increment τ in contrast to the exponential time increments generated in the SSA. To control the loss of accuracy with this approximation, the choice of leap-size τ must satisfy the historically referenced "leap condition" which is large enough that many events occur in that time, but nevertheless small enough that the intensity function value r is likely to change only "infinitesimally" as a consequence of those events. To the extent that this condition is satisfied, the mathematical rationale in replacing Markovian kinetics with Poisson kinetics [22] states that the number of times each independent event j will occur in the set time length τ can be approximated by a Poisson random variable with mean $\omega(t, t+\tau)$ on the interval $(t, t+\tau)$. For the ordinary τ-leap scheme, we assign $\omega(t, t+\tau) = r_j(X_t)\tau$. Thus, we set the intensity of event j equal to the constant $r_j = r_j(X_t)$ and we update the cell count vector $X_{t+\tau} = X_t + \sum_{j=1}^{3} n_j v_j$, where n_j are independent Poisson variates with means $r_j\tau$. Beyond ordinary τ-leaping, advancements have been made in improving accuracy when anticipating changes in the various components of the intensity vector by expanding $r_j(X_{t+\tau})$ in a Taylor series around time t with base value $r_j(X_t)$ to derive linear and quadratic approximations [23].

Selection of τ increments

As mentioned previously, we first set a number K (e.g., $K = 1000$) SSA steps to perform very quickly at the initiation of the P clone (first initiated cell asymmetrically divides from pre-initiated cell) in order to exactly simulate the small clones and capture the early extinction stochastic event correctly. Then, if a P clone is still growing, we switch to a τ-leaping algorithm

to speed up the simulation of the larger clones without loss of much accuracy. In search of a balance between computational efficiency and accuracy for our hybrid SSA/τ-leap algorithm, we would like to take advantage of the leap condition by employing τ-leaping when the P clones are large, which will take a very large number of reaction events to change the intensity function "significantly", and the exact SSA when τ is required to be small so that only a few reactions would be leaped over regardless. Recent work by Sehl et al. [23, 24] and others derived and applied methods to anticipate the largest τ such that the leap condition will be satisfied and accuracy will not be undesirably diminished. This will require us to introduce a small positive constant ϵ, which must be chosen empirically, to denote the acceptable relative change in the intensity function vector r. Adhering to the results of Cao et al. [25], we then chose our increment to be the largest τ such that

$$\left|\frac{d}{dt}r_j(X_t)\right|\tau \leq \epsilon r_j(X_t) \quad \Rightarrow \quad \tau \leq \frac{\epsilon}{\alpha_P - \beta_P} \tag{26}$$

holds for all j. Further, Cao et al. [20] explore the problem of negative population sizes which may occur with some probability within the τ-leaping method. In the setup described, this happens extremely rarely since τ-leaping usually only begins for clones with a substantial number of cells that has a low probability of extinction because they are entering the exponential mean growth phase (described in more detail in the following section). Thus, we can reject this choice of τ that produced a negative size and reduce τ, by 1/2 for example, until no negative populations are produced since this is a rare event and will not impede our computational runtime [20].

To obtain better accuracy without compromising speed in simulation time, a recent step anticipation leap (SAL) method has been developed that generalizes the ordinary τ-leaping method by projecting linear and quadratic changes in reaction propensities [23]. However, due to the nature of the birth-death-mutation processes modeled for premalignant P clones in the MSCE-EAC hybrid simulation, the leap condition for all these methods produces a restraint on τ that does not depend on the current size of the clone, as seen in Eq. (26). The linear and quadratic extrapolations of the propensity functions do not yield major improvements in accuracy when τ does not depend on the clone size at time t. Therefore, we employ the ordinary τ-leaping scheme in which we set time length $\tau = \frac{\epsilon}{\alpha_P - \beta_P}$, choose ϵ empirically to obtain desirable accuracy with our choose of cellular kinetic parameters, and approximate the number of times each independent event j (either birth, death/differentiation, or mutation) will occur by a Poisson random variable with mean $\omega(t, t+\tau) = r_j(X_t)\tau$ on the interval $(t, t+\tau)$. We may apply the result of the following section that an independent b-d-m process produces a shifted geometric size distribution for non-extinct clones, given by Eq. (34) but with P clone parameters, and enjoys mean exponential growth with rate $\alpha_P - \beta_P$. See Q-Q plot comparison in S3 Figure of both SSA and τ-leap algorithms against the true theoretical shifted geometric distribution for the b-d-m process. In practice with the estimated parameters given in Table 1, the hybrid SSA/τ-leap algorithm utilizes small values of $\tau < .004$ years, which allows even more accuracy yet still benefits from far less computational time than if we were to use solely SSA type steps.

Malignant size distribution

Expanding on Step 5 of the MSCE-EAC algorithm above, when a malignant progenitor M cell is born at time τ_3, an independent birth-death-detection process begins and we have

the analytical solution to the corresponding Kolmogorov equation for the generating function $\Phi_M(y_3, z; \tau, t)$ (from Eq. (2)) as derived in Eq. (3.5) of Jeon et al. [18]. Thus, we can make use of previous theoretical results here allowing us to avoid further simulation. We are first interested in knowing whether this malignant clone born at time τ_3 leads to a clinical EAC by time t_s. To generate this potential outcome, we use the 1-stage survival function, where $u = t_s - \tau_3$

$$S_M(u) \equiv \Pr[D(t_s) = 0 | M(\tau_3) = 1] = \Phi_M(1, 0; \tau_3, t_s) = 1 + \frac{1}{\alpha_M} \frac{p_M q_M e^{-p_M u} - q_M p_M e^{-q_M u}}{q_M e^{-p_M u} - p_M e^{-q_M u}} \quad (27)$$

with

$$p_M = \frac{1}{2}(-(\alpha_M - \beta_M - \rho) - \sqrt{(\alpha_M - \beta_M - \rho)^2 + 4\alpha_M \rho}) \quad (28)$$

$$q_M = \frac{1}{2}(-(\alpha_M - \beta_M - \rho) + \sqrt{(\alpha_M - \beta_M - \rho)^2 + 4\alpha_M \rho}) \quad (29)$$

Letting $p = 1 - S_M(t_s - \tau_3)$ be the probability of cancer detection of a particular malignant clone born at time τ_3, we first draw a Bernoulli random variable with probability p to decide if this clone will be detected as a clinical EAC by time t_s. The algorithm draws Bernoulli deviates from this 1-stage survival for each malignant clone in a BE patient and provides an EAC detection prevalence by time t_s.

For a patient in whom a malignant clone born at time τ_3 does not result in clinical EAC by time t_s, we would now like to use an analytical distribution to generate the size of the malignant clone present at time t_s, conditional that it did not undergo EAC detection. Jeon et al. [18] derived this conditional size distribution for a birth-death-detection process, which turns out in fact to be a shifted geometric distribution. Following Eqs.(3.9-3.16) in that paper, we have the size distribution of a malignant clone, given that no clinical cancer develops by time t_s

$$P_0 \equiv \Pr[M(\tau_3, t_s) = 0 | D(\tau_3, t_s) = 0, M(\tau_3, \tau_3) = 1] \quad (30)$$

$$= \frac{\zeta_M(\alpha_M + p_M)(\alpha_M + q_M)(q_M e^{-p_M u} - p_M e^{-q_M u})}{q_M(\alpha_M + p_M)e^{-p_M u} - p_M(\alpha_M + q_M)e^{-q_M u}} \quad (31)$$

and for $n \geq 1$,

$$P_n \equiv \Pr[M(\tau_3, t_s) = n | D(\tau_3, t_s) = 0, M(\tau_3, \tau_3) = 1] \quad (32)$$

$$= \frac{1}{n!} \frac{\partial^n \Phi_M(y_3, 0; \tau_3, t_s)}{\partial y_3^n}\bigg|_{y_3=0} \frac{1}{\Phi_M(1, 0; \tau_3, t_s)} \quad (33)$$

$$= (1 - P_0)(1 - \alpha_M \zeta_M)(\alpha_M \zeta_M)^{n-1} \quad (34)$$

where

$$\zeta_M = \frac{e^{-p_M u} - e^{-q_M u}}{(q_M + \alpha_M)e^{-p_M u} - (p_M + \alpha_M)e^{-q_M u}}$$

Thus, conditional on a malignant clone remaining undetected by time t_s, we again construct an inverse cumulative function and begin with a uniform random deviate $u \in [0, 1]$. If $P_0 \geq u$,

this particular malignant clone in question goes extinct before time t_s. If $u > P_0$, we derive the inverse cumulative function as follows

$$\begin{aligned}
\Pr[M(u) > n] &= \sum_{k=n+1}^{\infty} \Pr{}^*[M(u) = k] = \sum_{k=n+1}^{\infty} (1 - P_0)(1 - \alpha_{_M}\zeta_{_M})(\alpha_{_M}\zeta_{_M})^{k-1} \\
&= (1 - P_0)(1 - \alpha_{_M}\zeta_{_M})(\alpha_{_M}\zeta_{_M})^{n} \sum_{k=n+1}^{\infty} (\alpha_{_M}\zeta_{_M})^{k-(n+1)} \\
\Rightarrow \Pr[M(u) \leq n] &= 1 - (1 - P_0)(\alpha_{_M}\zeta_{_M})^{n}
\end{aligned}$$

Thus, we may generate a size n from this distribution,

$$n = \frac{\ln\left(\dfrac{1-u}{1-P_0^*}\right)}{\ln(\alpha_{_M}\zeta_{_M})} \quad \text{with } u > P_0^*$$

Example simulation details

For the Results presented in the Main Text, we simulated an index endoscopy for all males and females age $t_s = 60$ in the year 1990 (indicative of index screens from prospective studies that estimate the BE to EAC progression rate). With BE prevalence F_{BE} given in Eq. (24), the Results focus on output regarding the subpopulation of individuals found with BE, for whom the MSCE-EAC screening model obtains screening results (see Methods).

We generated a BE segment length for each patient from a beta distribution with shape parameters 16/11 and 4, restricted to the range of 1-16 cm for both males and females. BE segments of the simulated patients have an average length of 4.9 cm. Short segment BE (less than 3 cm) comprises 22% of the density and long segment BE (greater than 3 cm) comprises 78%. This BE distribution recreates the proportions of long and short segments recorded for the study patient population in [12]. S4-S7 Figures depict the number distributions and long-tailed, Luria-Delbruck type size distributions for the non-extinct premalignant and malignant clones, respectively, present at time $t_s = 60$ for the cohorts of males and females separately. Based on 100K simulation, the mean number of premalignant clones per BE patient, without symptomatic cancer, in this cohort is 6.6, while only about 1% of these dysplastic clones harbor a non-extinct malignancy.

MSCE-EAC screening module: EAC incidence projections

Here we derive all components of the general cumulative hazard $\Lambda_{_{MSCE}}(t)$, given by

$$\Lambda_{_{MSCE}}(t) = -\ln(S_{_{MSCE}}(t)) = -\ln\left(1 - \int_0^t f_{_{MSCE}}(s)\,\mathrm{d}s\right) \tag{35}$$

For the initial scenario of screening all individuals at time t_s, we derived the MSCE-EAC cumulative hazard function that includes contributions from the subpopulation of those individuals found to have BE at time t_s that, immediately following HGD diagnosis, may receive treatment at time t_s; and the subpopulation without BE. For any time $t > t_s$ and BE cumulative density

function F_{BE} given in Eq. (24), we compute the MSCE-EAC density function $f_{MSCE}(s)$ for the general population explicitly as follows

$$f_{MSCE}(s) = f_{MSCE}(s|T_{BE} \leq t_s) \cdot \Pr[T_{BE} \leq t_s] + f_{MSCE}(s|T_{BE} > t_s) \cdot \Pr[T_{BE} > t_s] \tag{36}$$

The convolution formula for the unscreened population contributing to the MSCE-EAC density is given by

$$f_{MSCE}(t|T_{BE} > t_s) = \frac{1}{\Pr[T_{BE} > t_s]} \int_{t_s}^{t} f_{BE}(u) f_{4/MS}(t-u)\,\mathrm{d}u \tag{37}$$

$$= e^{\int_0^{t_s} \nu(s)\,\mathrm{d}s} \cdot \int_{t_s}^{t} \nu(u) e^{-\int_0^u \nu(s)\,\mathrm{d}s} \cdot f_{4/MS}(t-u)\,\mathrm{d}u \tag{38}$$

where $f_{4/MS}(t-u) = -Y_8(t-u, t-u)$ (see Eq. (19)) is the numerical solution for the 4-stage multistage model after BE onset.

For the screened BE population, we follow the method of Jeon et al. [18] and consider the 4 possible types of cells present in a patient at screening time t_s^- (where the minus superscript denotes cell populations present prior to any intervention) X = number of BE stem cells in BE segment, $P^*(t_s^-)$ = number of pre-initiated P^* cells, $P(t_s^-)$ = number of initiated, dysplastic P cells (all clones combined), $M(t_s^-)$ = number of malignant, preclinical cells (all clones combined). The MSCE-EAC hybrid simulation records these random variables for each BE patient at the instance of screening t_s^-, before any intervention occurs. After simulating n independent and identically distributed (by gender) individuals and performing the Seattle biopsy screening protocol *in silico* as described in Methods, the simulation obtains the vector $A_i = \{X_i, P_i^*(t_s^-), P_i(t_s^-), M_i(t_s^-)\}$ for each patient with BE, $i = 1, .., n$.

As described in the Main Text, we explore the simulated intervention of RFA of dysplasia patients by introducing the following ablation proportion vector, $\omega = \{\omega_X, \omega_{P*}, \omega_P, \omega_M\}$, to deplete the existing cell types and leave a specified fraction (based on desired effectiveness of RFA treatment) in the esophagus given by ω. We adjust each dysplastic patient's cell count vector A_i through component-wise multiplication by ω. Thus, the post-RFA numbers of cells in each stage of the MSCE process, in simulated patient i, immediately after screening and treatment (denoted by time t_s^+) are given by an adjusted cell type vector $\hat{A}_i$ as follows

$$\hat{A}_i \equiv \omega \circ A_i = \{\omega_X \cdot X_i, \omega_{P*} \cdot P_i^*(t_s^-), \omega_P \cdot P_i(t_s^-), \omega_M \cdot M_i(t_s^-)\} \tag{39}$$

$$= \{X_i(t_s^+), P_i^*(t_s^+), P_i(t_s^+), M_i(t_s^+)\} \tag{40}$$

$$\equiv \{\hat{X}_i, \hat{P}_i^*, \hat{P}_i, \hat{M}_i\} \tag{41}$$

BE patients with a negative screen for dysplasia sustain the same (before and after) $A_i \equiv \hat{A}_i$ vector as was computed at time t_s^- since no RFA treatment is performed on these patients. Due to Markovian renewal of the branching process, we may then compute the survival and hazard functions, as in [18], for each screened individual $i = 1, .., n$ for some time $t > t_s$ with

contributions from each cell type post screen

$$S_{MSCE}(t-t_s|\hat{A}_i) = S_4(t-t_s)^{\hat{X}_i} S_3(t-t_s)^{\hat{P}_i^*} S_2(t-t_s)^{\hat{P}_i} S_1(t-t_s)^{\hat{M}_i} \tag{42}$$

$$h_{MSCE}(t-t_s|\hat{A}_i) = \hat{X}_i h_4(t-t_s) + \hat{P}_i^* h_3(t-t_s) + \hat{P}_i h_2(t-t_s) + \hat{M}_i h_1(t-t_s) \tag{43}$$

$$\Rightarrow \quad f_{MSCE}(t|T_{BE} \leq t_s) \approx \frac{1}{n} \sum_{j=1}^{n} h(t-t_s|\hat{A}_j) \cdot S(t-t_s|\hat{A}_j) \tag{44}$$

These survival and hazard functions for the 4-stage model after BE onset may be easily computed incorporating the Kolmogorov backward equations. The 8 ODEs from Eqs.(12-19) can be solved numerically to obtain the survival and hazard functions we require

$$h_4(t-t_s) = -\frac{Y_8(t-t_s)}{Y_7(t-t_s)} \quad \text{and} \quad S_4(t) = Y_7(t-t_s),$$

$$h_3(t-t_s) = -\frac{Y_6(t-t_s)}{Y_5(t-t_s)} \quad \text{and} \quad S_3(t) = Y_5(t-t_s),$$

$$h_2(t-t_s) = -\frac{Y_4(t-t_s)}{Y_3(t-t_s)} \quad \text{and} \quad S_2(t) = Y_3(t-t_s),$$

$$h_1(t-t_s) = -\frac{Y_2(t-t_s)}{Y_1(t-t_s)} \quad \text{and} \quad S_1(t) = Y_1(t-t_s).$$

We have now derived all necessary components of $\Lambda_{MSCE}(t)$ of Eq. (35) after a single screen of all individuals at time t_s. See the Results section for an illustrative figure.

Open source code

The method outlined in this section is implemented by the comprehensive MSCE-EAC screening model consisting of three modules: cell, tissue, and screening. All necessary tools to employ this method, including examples of user inputs used in the upcoming Results, are available in documented R code at `https://github.com/yosoykit/MSCE_EAC_Screening_Model`.

References

1. Moolgavkar S, Dewanji A, Venzon D (1988) A stochastic two-stage model for cancer risk assessment. ii. the number and size of premalignant clones. the hazard function and the probability of tumor. Risk Anal 8: 383-392.

2. Little M (1995) Are two mutations sufficient to cause cancer? Some generalizations of the two-mutation model of carcinogenesis of Moolgavkar, Venzon, and Knudson, and of the multistage model of Armitage and Doll. Biometrics 51: 1278-1291.

3. Luebeck E, Moolgavkar S (2002) Multistage carcinogenesis and the incidence of colorectal cancer. Proc Natl Acad Sci U S A 99: 15095-15100.

4. Crump C, Subramaniam R, Van Landingham C (2005) A numerical solution to the non-homogeneous two-stage mvk model of cancer. Risk Anal 25: 921-926.

5. Kong CY, Kroep S, Curtius K, Hazelton WD, Jeon J, et al. (2014) Exploring the recent trend in esophageal adenocarcinoma incidence and mortality using comparative simulation modeling. Cancer Epidemiol Biomarkers Prev 23: 997-1006.

6. Ruigomez A, Wallander M, Lundborg P, Johansson S, Garcia Rodriguez L (2010) Gastroesophageal reflux disease in children and adolescents in primary care. Scan J Gastroenterology 45: 139-146.

7. Ruigomez A, Garcia Rodriguez L, Wallander M, Johansson S, Graffner H, et al. (2004) Natural history of gastro-oesophageal reflux disease diagnosed in general practice. Aliment Pharmacol Ther 20: 751-760.

8. Dent J, El-Serag H, Wallander M, Johansson S (2005) Epidemiology of gastro-oesophageal reflux disease: a systematic review. Gut 54: 710–717.

9. Taylor J, Rubenstein J (2010) Meta-analyses of the effect of symptoms of gastroesophageal reflux on the risk of Barrett's esophagus. Am J Gastroenterol 105: 1730-1737.

10. Surveillance, Epidemiology, and End Results (SEER) Program (www.seer.cancer.gov) SEER*Stat Database: Incidence - SEER 9 Regs Research Data, Nov 2013 Sub (1973-2011) Katrina/Rita Population Adjustment - Linked To County Attributes - Total U.S., 1969-2012 Counties, National Cancer Institute, DCCPS, Surveillance Research Program, Surveillance Systems Branch, released April 2014, based on the November 2013 submission.

11. Heidenreich W, Luebeck E, Moolgavkar S (1997) Some properties of the hazard function of the two-mutation clonal expansion model. Risk Anal 17: 391-399.

12. O'Connor J, Falk G, Richter J (1999) The incidence of adenocarcinoma and dysplasia in Barrett's esophagus report on the cleveland clinic barrett's esophagus registry. Am J Gastroenterol 94: 2037-2042.

13. Conio M, Blanchi S, Lapertosa G, Ferraris R, Sablich R, et al. (2003) Long-term endoscopic surveillance of patients with Barrett's esophagus. incidence of dysplasia and adenocarcinoma: A prospective study. Am J Gastroenterol 98: 1931-1939.

14. Sharma P, Falk G, Weston A, Reker D, Johnston M, et al. (2006) Dysplasia and cancer in a large multicenter cohort of patients with Barrett's esophagus. Clin Gastroeneterol H 4: 566-572.

15. Miros M, Kerlin P, Walker N (1991) Only patients with dysplasia progress to adenocarcinoma in Barrett's oesophagus. Gut 32: 1441-1446.

16. Guardino J, Khandwala F, Lopez R, Wachsberger D, Richter J, et al. (2006) Barrett's esophagus at a tertiary care center: Association of age on incidence and prevalence of dysplasia and adenocarcinoma. Am J Gastroenterol 101: 2187-2193.

17. Weston A, Badr A, Hassanein R (1999) Prospective multivariate analysis of clinical, endoscopic, and histological factors predictive of the development of Barrett's multifocal high-grade dysplasia or adenocarcinoma. Am J Gastroenterol 94: 3413-3419.

18. Jeon J, Meza R, Moolgavkar S, Luebeck G (2008) Evaluation of screening strategies for pre-malignant lesions using a biomathematical approach. Math Biosci 213: 56-70.

19. Gillespie D (2001) Approximate accelerated stochastic simulation of chemically reacting systems. J Chem Phys 115: 1716–1733.

20. Cao Y, Gillespie D, Petzold L (2005) Avoiding negative populations in explicit poisson tau-leaping. J Chem Phys 123.

21. Gillespie D, Petzold L (2003) Improved leap-size selection for accelerated stochastic simulation. J Chem Phys 119: 8229–8234.

22. Kurtz T (1981) Approximation of Population Processes, Philadelpha, PA: SIAM.

23. Sehl M, Alekseyenko A, Lange K (2009) Accurate stochastic simulation via step anticipation τ-leaping (SAL) algorithm. J Comput Bio 16: 1195–1208.

24. Sehl M, Zhou H, Sinsheimer J, Lange K (2011) Extinction models for cancer stem cell therapy. Math Biosci 234: 32-146.

25. Cao Y, Gillespie D, Petzold L (2006) Efficient step size selection for the tau-leaping simulation method. J Chem Phys 124.
